# Rotavirus-Induced Early Activation of the RhoA/ROCK/MLC Signaling Pathway Mediates the Disruption of Tight Junctions in Polarized MDCK Cells

**DOI:** 10.1038/s41598-018-32352-y

**Published:** 2018-09-17

**Authors:** Mahmoud Soliman, Eun-Hyo Cho, Jun-Gyu Park, Ji-Yun Kim, Mia Madel Alfajaro, Yeong-Bin Baek, Deok-Song Kim, Mun-Il Kang, Sang-Ik Park, Kyoung-Oh Cho

**Affiliations:** Laboratory of Veterinary Pathology, College of Veterinary Medicine, Chonnam National University, Gwangju, Republic of Korea

## Abstract

Intestinal epithelial tight junctions (TJ) are a major barrier restricting the entry of various harmful factors including pathogens; however, they also represent an important entry portal for pathogens. Although the rotavirus-induced early disruption of TJ integrity and targeting of TJ proteins as coreceptors are well-defined, the precise molecular mechanisms involved remain unknown. In the present study, infection of polarized MDCK cells with the species A rotavirus (RVA) strains human DS-1 and bovine NCDV induced a redistribution of TJ proteins into the cytoplasm, a reversible decrease in transepithelial resistance, and an increase in paracellular permeability. RhoA/ROCK/MLC signaling was identified as activated at an early stage of infection, while inhibition of this pathway prevented the rotavirus-induced early disruption of TJ integrity and alteration of TJ protein distribution. Activation of pMYPT, PKC, or MLCK, which are known to participate in TJ dissociation, was not observed in MDCK cells infected with either rotavirus strain. Our data demonstrated that binding of RVA virions or cogent VP8* proteins to cellular receptors activates RhoA/ROCK/MLC signaling, which alters TJ protein distribution and disrupts TJ integrity via contraction of the perijunctional actomyosin ring, facilitating virion access to coreceptors and entry into cells.

## Introduction

The gastrointestinal epithelium consists of a multitude of cell types and acts as a selective barrier that prevents potentially harmful luminal agents, such as microorganisms and their products, food antigens, or toxins from penetrating underlying tissues, while allowing for the exchange of ions and small molecules^1^. This barrier function is achieved through cell-cell contacts between adjacent cell membranes. Tight junctions (TJs), the most apical component of the apical junctional complex, which also include adherens junctions and desmosomes, have a key role in this barrier function. TJs seal the epithelium, maintain tissue integrity, and demarcate the boundary between the apical and basolateral plasma membrane^1,2^. TJ transmembrane proteins are often grouped according to the number of times they span the plasma membrane; for example, the single-pass junctional adhesion molecules (JAMs) as well as coxsackievirus and adenovirus receptor (CAR) proteins, the three-pass blood vessel/epicardial substance, and the four-pass claudin, occludin, MarvelD3, and tricellulin proteins^3^. Although the majority of TJ proteins have at least some adhesive abilities, the four-pass membrane proteins exert more direct epithelial barrier functions^3^. The intracellular domains of these transmembrane proteins interact with cytosolic scaffold proteins, such as zonula occludens (ZO), which in turn link these transmembrane proteins to the actin cytoskeleton^4–6^.

TJ dissociation results in a decrease in transepithelial electrical resistance (TER) and an increase in paracellular permeability^1,7^, leading to various diseases, such as inflammatory bowel disease, vasogenic edema, and cancers^2,8–10^. Many viruses disrupt TJs to access the buried basolateral proteins under these structures, which they co-opt as attachment and entry receptors^1,2,6,11^. The key mechanisms involved in virus-induced early disruption of TJs include activation of host cell signaling pathways via binding of virus particles to their primary receptors, reorganization or degradation of specific TJ proteins, and/or contraction of the perijunctional actomyosin ring (formed from stress fibers)^1,2,6,11^. The assembly and disassembly of TJs are exquisitely orchestrated by the interaction of various signaling molecules such as those in the RhoA, protein kinase C (PKC), PKA, myosin light chain kinase (MLCK), mitogen-activated protein kinase (MAPK), phosphatase, and phosphoinositide 3-kinase signaling pathways^2,4,6,12^. Among these signaling pathways, RhoA and its downstream effector Rho kinase (ROCK) as well as PKC and its downstream effector MLCK are crucial in mediating TJ dissociation; this can be mediated through direct phosphorylation of the myosin II regulatory light chain (MLC) or indirectly through inhibition of dephosphorylation of MLC via activation of the regulatory subunit of myosin light chain phosphatase (MYPT), providing the force for disruption of TJs upon contraction of the perijunctional actomyosin ring^4,13^.

Species A rotaviruses (RVAs), members of the *Rotavirus* genus in the *Reoviridae* family, are a major cause of pediatric diarrhea worldwide and are responsible for approximately 200,000 deaths of children under the age of 5 years annually^14,15^. RVAs also cause severe acute dehydrating diarrhea in a wide variety of young animals, resulting in significant economic losses^16^. RVAs are triple-layered particles (TLPs) that contain 11 segments of genomic double-stranded RNA (dsRNA), encoding six structural (VP1–4, VP6, and VP7) and six non-structural proteins (NSP1–NSP6)^17,18^. The outermost layer of virion is composed of two proteins, the spike protein VP4 and the glycoprotein VP7; VP4 is cleaved into two fragments, VP8* and VP5* by trypsin^19,20^. RVA surface proteins interact with different cell surface receptors to enter cells via a complex multistep process^21,22^. Following sequential virus binding to a receptor and a co-receptor, most RVAs enter the cell by clathrin-mediated endocytosis^21,23,24^, although some RVAs, such as rhesus rotavirus (RRV), enter cells via a clathrin- and caveolin-independent pathway^21,25,26^.

RVAs primarily infect mature enterocytes of the small intestine, although there is possibility of infection of extraintestinal tissues^27–31^, while a variety of cells of epithelial origin in culture are highly permissible for RVA infection^17^. Recent data from a genome-wide RNAi screen indicated that JAM-A, occludin, and ZO-1 play important roles during rotavirus entry into MA104 cells^26^. Subsequently, it was observed that JAM-A functions as a coreceptor for rotavirus in MA104 cells, and that occludin and ZO-1 are also required during the entry process^32^. For RVAs to access TJ proteins such as JAM-A, it is necessary for TJs to be unwound. In polarized epithelial cells (e.g., MDCK cells), RVA infection induces a drop in TER and an increase in paracellular flux via dissociation of TJs, which is induced by specific binding of the VP8* protein to its cellular receptors^7^. RVA infection induces the activation of RhoA GTPase and the formation of stress fibers, resulting in disruption of the cell cytoskeleton^33^; however, the specific involvement of cellular signaling pathways, in particular RhoA/ROCK and its associated PKC signaling pathway, in RVA-induced TJ disruption, has remained elusive.

The present study addresses the mechanisms by which RVA modulates TJ integrity to enter polarized epithelial cells. We demonstrate that RVA infection induces phosphorylation of MLC via the RhoA/ROCK signaling pathway, independently of PKC/MLCK signaling. Activation of RhoA/ROCK/MLC signaling results in contraction of the actomyosin ring and an increase in TJ permeability. Inhibition of the RhoA/ROCK/MLC signaling pathway using various compounds restores TER and prevents RVA infection-induced TJ permeability in polarized epithelial cells.

## Results

### Rotavirus induces expression of pMLC via the RhoA/ROCK signaling pathway in polarized epithelial cells

**Figure 1:**
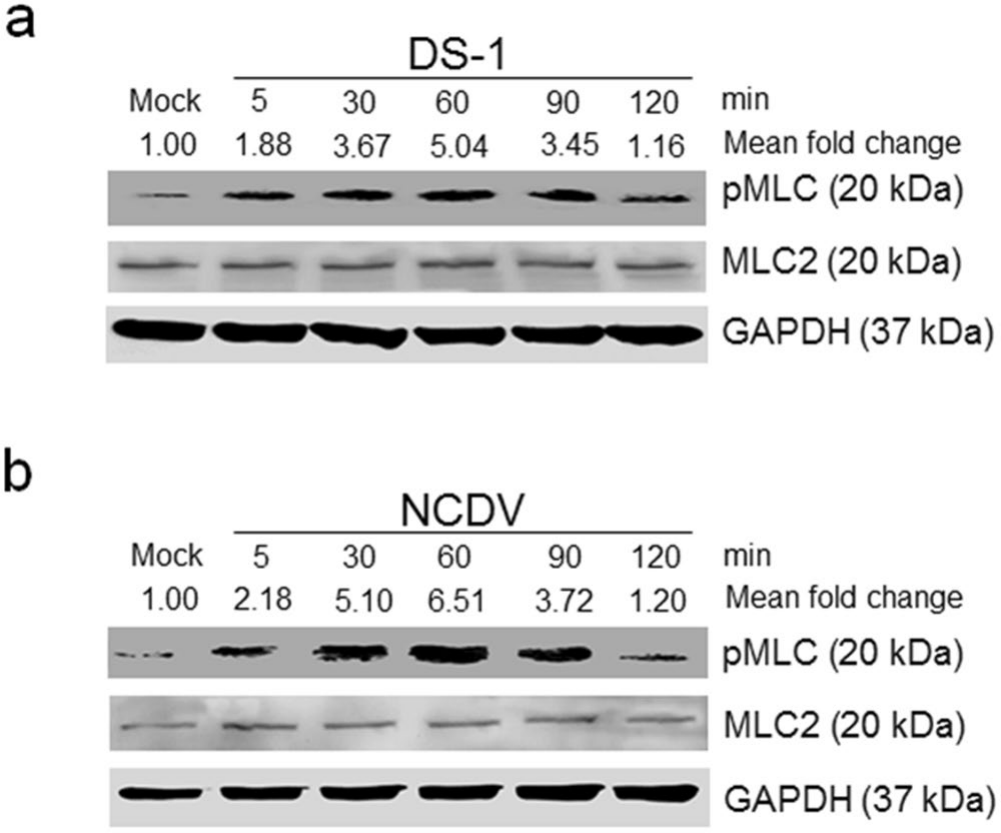
Rotavirus infection-induced phosphorylation of MLC in polarized epithelial cells is an early event. (**a** and **b**) Human RVA DS-1 and bovine RVA NCDV strains (MOI = 10) were inoculated into confluent MDCK monolayers, and cells were harvested at the indicated time points. Cell lysates were subjected to western blot analysis to determine expression levels of phosphorylated MLC (pMLC) and MLC2 using the relevant antibodies. GAPDH was used as a loading control. The intensity of pMLC relative to GAPDH was determined by densitometric analysis and is indicated above each lane. All experiments were performed in triplicate and representative images of different gels from each group are presented.

Phosphorylation of MLC is important for TJ disruption via actomyosin contraction^4,13^. To determine whether early-infection with RVA induces phosphorylation of MLC, confluent MDCK cells were either mock-infected or infected with the human RVA strain (DS-1) or the bovine strain (NCDV) at a multiplicity of infection (MOI) of 10. Western blot analysis of cell lysates harvested at the indicated time points was performed using a specific antibody against pMLC (Ser19). In contrast to mock-infected MDCK cells, in which pMLC remained unchanged during the entire experiment, infection with either RVA strain resulted in a remarkable increase in pMLC that was detected as early as 5 min post-infection (mpi), peaked at 30 mpi, and was sustained at a high level until 60 mpi, declining afterwards (Fig. 1a,b).

**Figure 2:**
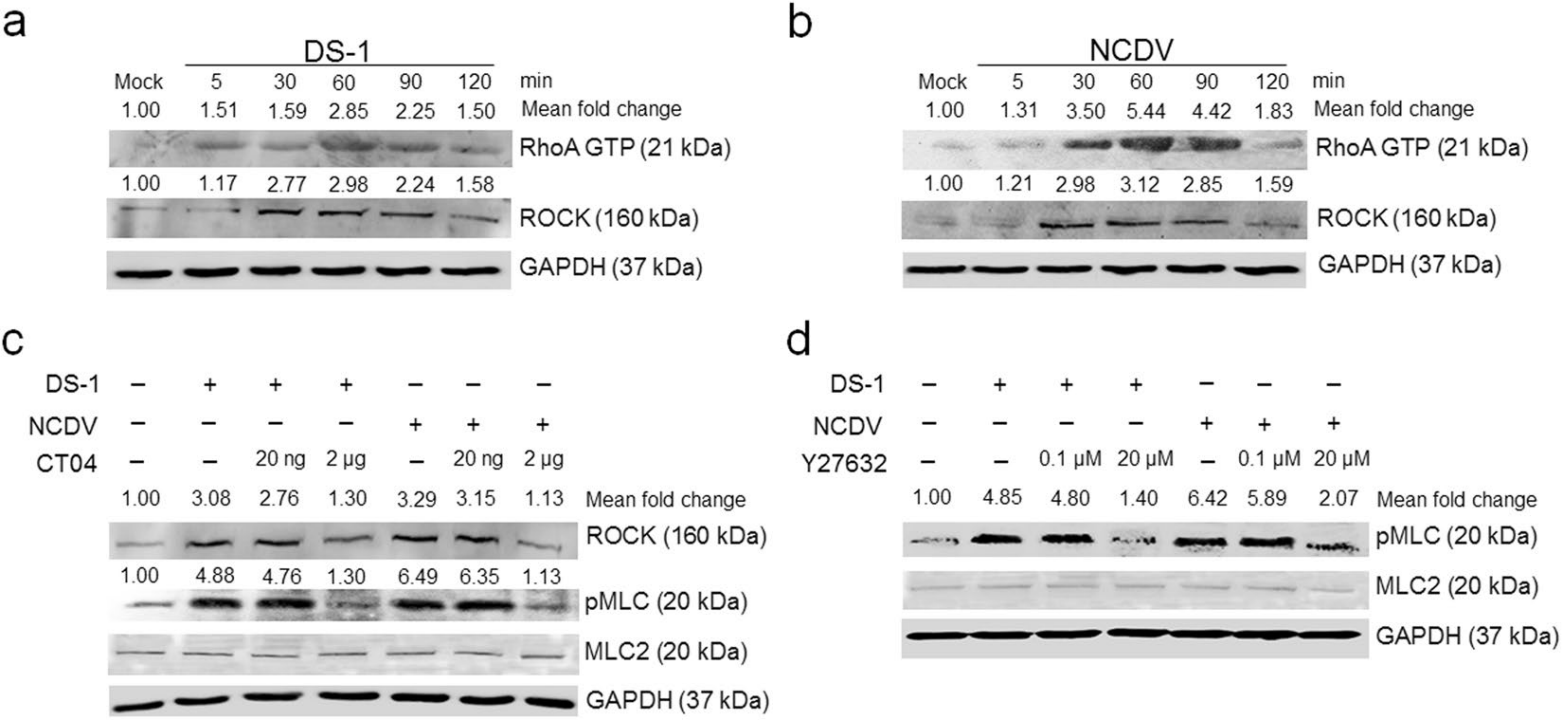
The RhoA/ROCK signaling pathway is involved in rotavirus-induced early activation of pMLC. (**a** and **b**) Confluent MDCK monolayers were mock-infected or infected with RVA strain (**a**) DS-1 or (**b**) NCDV (MOI = 10) for the indicated time points. The cells were then harvested and subjected to a Rhotekin pull-down assay to measure RhoA activation and then analyzed by western blot analysis using an anti-RhoA antibody or directly analyzed by western blot analysis to examine the expression levels of ROCK using specific primary antibody. (**c** and **d**) Confluent MDCK monolayers were pretreated with or without (**c**) RhoA inhibitor (CT04) or (**d**) ROCK inhibitor (Y27632) at the indicated doses for 1 h at 37 °C and then infected with strain DS-1 or NCDV (MOI = 10). Cell lysates were harvested at 1 h post-infection, and the expression levels of ROCK and/or pMLC were evaluated by western blot analysis. GAPDH was used as a loading control. The intensities of RhoA, ROCK, and pMLC relative to GAPDH were determined by densitometric analysis and are indicated above each lane. All experiments were performed in triplicate and representative images of different gels from each group are presented.

To identify the upstream effectors of pMLC in RVA-infected cells, confluent MDCK cells were infected at the indicated time points, and the resulting cell lysates were used in a pull-down assay using Rhotekin RBD agarose beads, followed by RhoA detection by western blot analysis using a specific anti-RhoA antibody. Alternatively, cell lysates were directly subjected to western blot analysis to investigate changes in ROCK expression using a specific antibody^34–37^. RVA infection induced the activation of RhoA and ROCK in confluent MDCK cells as early as 5 mpi, which peaked at 60 mpi and was sustained at high levels until 90 mpi, subsequently declining (Fig. 2a,b). To confirm these results, MDCK cells were pretreated for 1 h at 37 °C with non-cytotoxic concentrations of inhibitors specific to RhoA (CT04) or ROCK (Y27632) (Supplementary Fig. S1), incubated with either RVA strain for 1 h, and analyzed for activation of ROCK and/or pMLC by western blot analysis. ROCK expression levels were diminished following treatment with the RhoA inhibitor CT04, and pMLC levels were reduced after treatment with the RhoA inhibitor CT04 or ROCK inhibitor Y27632 in a dose-dependent manner (Fig. 2c,d). Taken together, these data suggest that pMLC is activated in RVA-infected cells in a RhoA/ROCK-dependent manner.

### RVA-induced early activation of pMLC is independent of RhoA/ROCK/MYPT and PKC/MLCK signaling pathways

ROCK can induce phosphorylation of MYPT (pMYPT) at residues Thr696 and Thr853, resulting in inhibition of myosin phosphatase and consequent hyperphosphorylation of MLC^4,13^. To determine whether RVA infection induces the phosphorylation of MYPT at early time points, confluent MDCK cells were infected and lysed at the indicated time points, and then pMYPT was detected by western blot analysis using specific anti-pMYPT or MYPT antibodies. Neither of the RVA strains (DS-1 and NCDV) triggered the phosphorylation of MYPT1 in confluent MDCK cells at any time point (Supplementary Fig. S2a,b). To confirm these results, confluent MDCK cells were either mock-infected or infected with DS-1 or NCDV, and cell lysates were harvested at the indicated time points and immunoprecipitated using antibodies specific for pMYPT or ROCK. Consistent with the western blot findings, neither antibody precipitated their specific antigen (Supplementary Fig. S2c,d), suggesting that pMLC phosphorylation occurred independently of the RhoA/ROCK/pMYPT pathway.

It has been previously established that the PKC/MLCK pathway affects the epithelial and endothelial barriers through MLC activation^4^. Therefore, we examined whether MLC phosphorylation is mediated through upregulation of the upstream PKC/MLCK signaling cascade^38–40^. Mock- or RVA-infected cell lysates were subjected to western blotting using antibodies against pPKC and MLCK. pPKC and MLCK levels were unchanged in virus-infected cells compared with mock-infected cells (Supplementary Fig. S3a,b). In addition, treatment of confluent MDCK cells with the PKC inhibitor Gö 6983 or the MLCK inhibitor ML7 before virus infection did not alter virus-activated pMLC levels (Supplementary Fig. S3c,d). These results suggest that both human and animal RVA strains can activate pMLC independently of PKC/MLCK signaling.

### Rotavirus outer capsid VP8* protein triggers activation of the RhoA/ROCK/MLC signaling pathway

**Figure 3:**
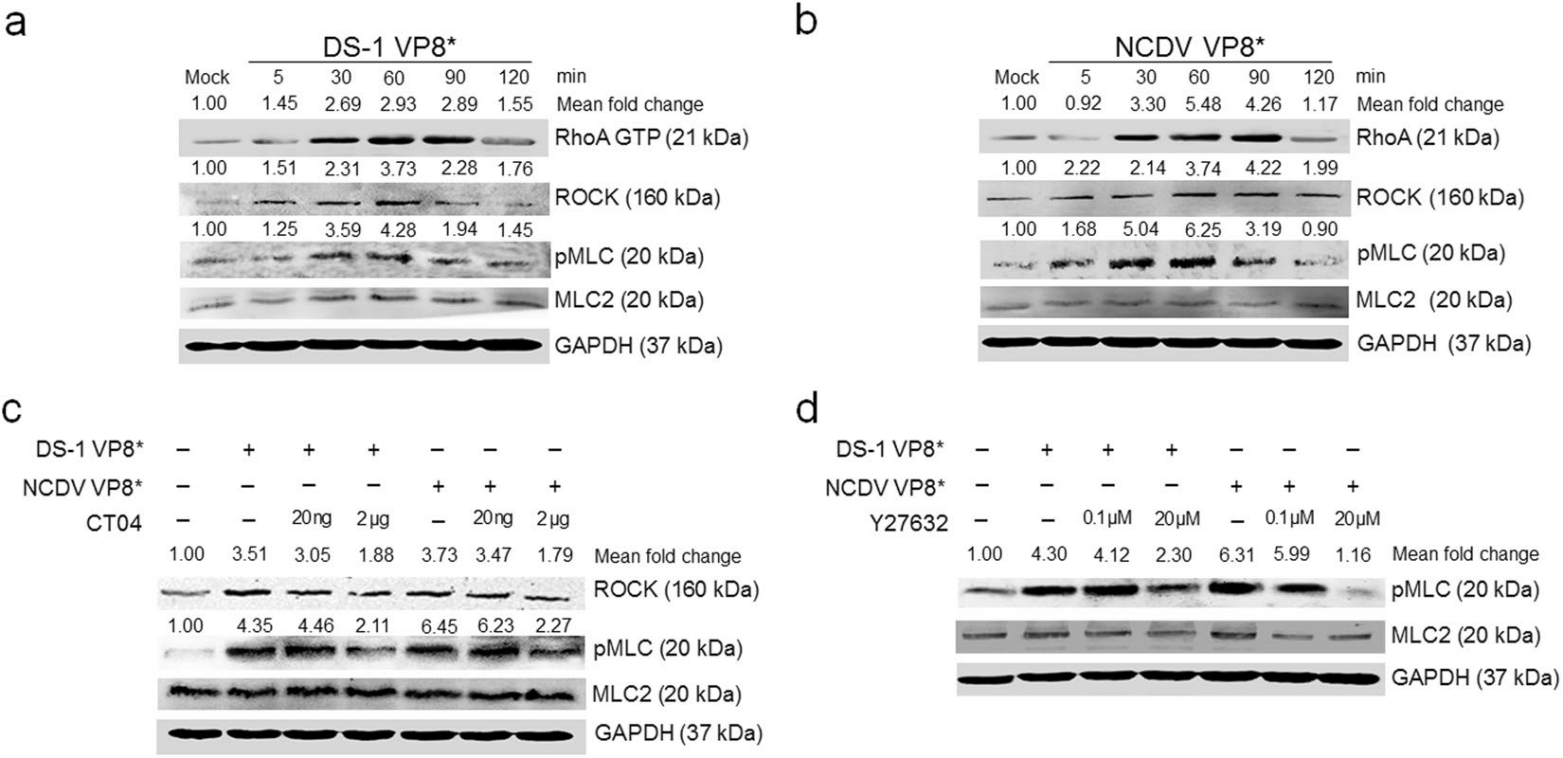
Rotavirus VP8* protein triggers activation of the RhoA/ROCK/MLC pathway. (**a** and **b**) Confluent MDCK monolayers were incubated with recombinant GST-tagged VP8* protein of RVA strain (**a**) DS-1 or (**b**) NCDV (10 μg/ml) for the indicated time points. Cell lysates were subjected to a Rhotekin pull-down assay to measure RhoA activation and then analyzed by western blot analysis using an anti-RhoA antibody or directly analyzed by western blot analysis to check the expression level of ROCK, pMLC, and MLC2 using the relevant antibodies. GAPDH was used as a loading control. (**c** and **d**) Confluent MDCK monolayers were either mock-treated or pretreated with (**c**) RhoA inhibitor CT04 or (**d**) ROCK inhibitor Y27632 for 1 h at 37 °C and then incubated with the purified VP8* protein of RVA strain DS-1 or NCDV (10 μg/ml). Cell lysates were harvested at 1 h and the expression levels of ROCK and/or pMLC were evaluated by western blot analysis using the relevant antibodies. GAPDH was used as a loading control. All experiments were performed in triplicate and representative images of different gels from each group are presented. The intensities of RhoA, ROCK, and pMLC relative to GAPDH were determined by densitometric analysis and are indicated above each lane.

The RVA outer capsid protein VP8* can impair TJ function and diminish the TER of polarized cell monolayers^7^. Therefore, we assessed whether recombinant RVA VP8* protein promotes the activation of RhoA/ROCK/MLC signaling. The addition of purified recombinant VP8* proteins (10 μg/ml) of RVA DS-1 and NCDV strains resulted in RhoA, ROCK, and pMLC activation in confluent MDCK cells (Fig. 3a,b). Moreover, pretreatment with RhoA inhibitor (CT04) reduced ROCK and pMLC levels, while the ROCK inhibitor (Y27632) reduced pMLC level in a dose-dependent manner (Fig. 3c,d). These data indicate that activation of RhoA/ROCK/MLC signaling is induced by cell receptor binding of rotavirus VP8* protein.

### Inhibition of the RhoA/ROCK/MLC signaling pathway reduces production of progeny viruses

**Figure 4:**
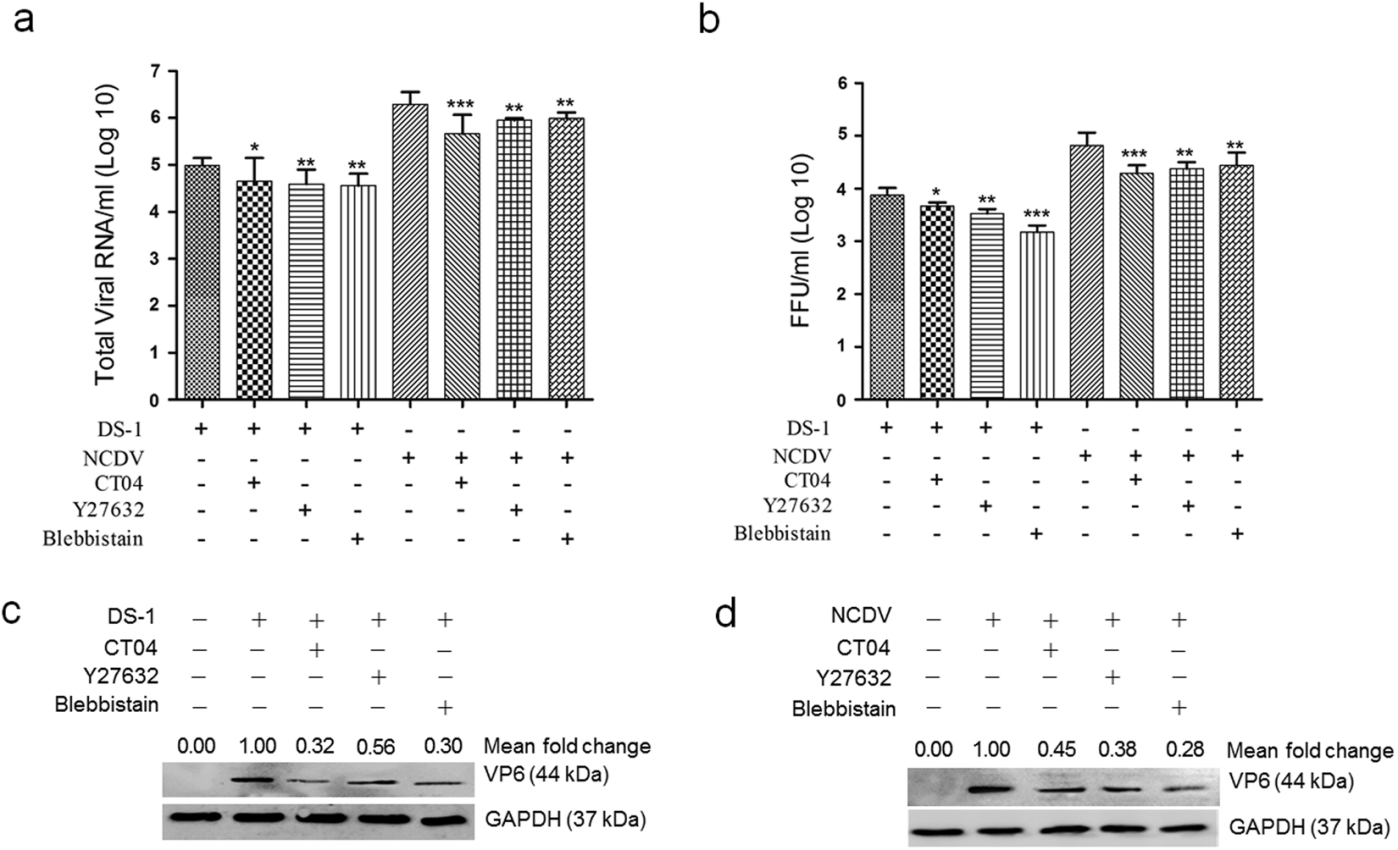
Inhibition of the RhoA/ROCK/MLC signaling pathway affects rotavirus infectivity and viral protein expression. MDCK cells were pretreated with non-cytotoxic concentrations of RhoA inhibitor (CT04), ROCK inhibitor (Y27632), or MLC inhibitor (blebbistatin) for 1 h at 37 °C and then infected with RVA strain DS-1 or NCDV (MOI = 10) for 12 h. (**a**) Total viral RNA was determined by real-time RT-PCR. (**b**) Virus titers were determined by cell culture immunofluorescence assay using cell lysates produced by three cycles of freezing and thawing; the results are expressed as fluorescent focus forming unit (FFU). (**c** and **d**) Viral VP6 protein was detected by western blot analysis. GAPDH was used as a loading control. The intensity of VP6 relative to GAPDH was determined by densitometric analysis and is indicated above each lane. All experiments were performed in triplicate. Representative images of different gels from each group are presented. Data are presented as the mean ± standard error of the mean from three independent experiments. Differences were evaluated using a one-way ANOVA. *p < 0.05; **p < 0.001; ***p < 0.0001.

Activation of the RhoA/ROCK/MLC signaling pathway during the early stage of RVA infection appears to be involved in the RVA life cycle; thus, blocking this pathway could influence virus replication. To further investigate whether these signaling molecules are involved in the RVA life cycle, we pretreated cells with specific inhibitors against RhoA (CT04), ROCK (Y27632), or MLC (blebbistatin) for 1 h, followed by RVA inoculation, incubation for 12 h, and infectivity measurement of the resulting viral progeny. Compared with the mock control, pretreatment with CT04, Y27632, or blebbistatin reduced total viral RNA levels (Fig. 4a), resulting in a decline in the infectivity of viral progeny (Fig. 4b) and viral protein expression (Fig. 4c,d). These findings suggest that early activation of RhoA/ROCK/MLC signaling during RVA infection affects production of progeny viruses.

### Rotavirus-induced alteration of transepithelial resistance in polarized epithelial cells is dependent on the RhoA/ROCK/MLC pathway

**Figure 5:**
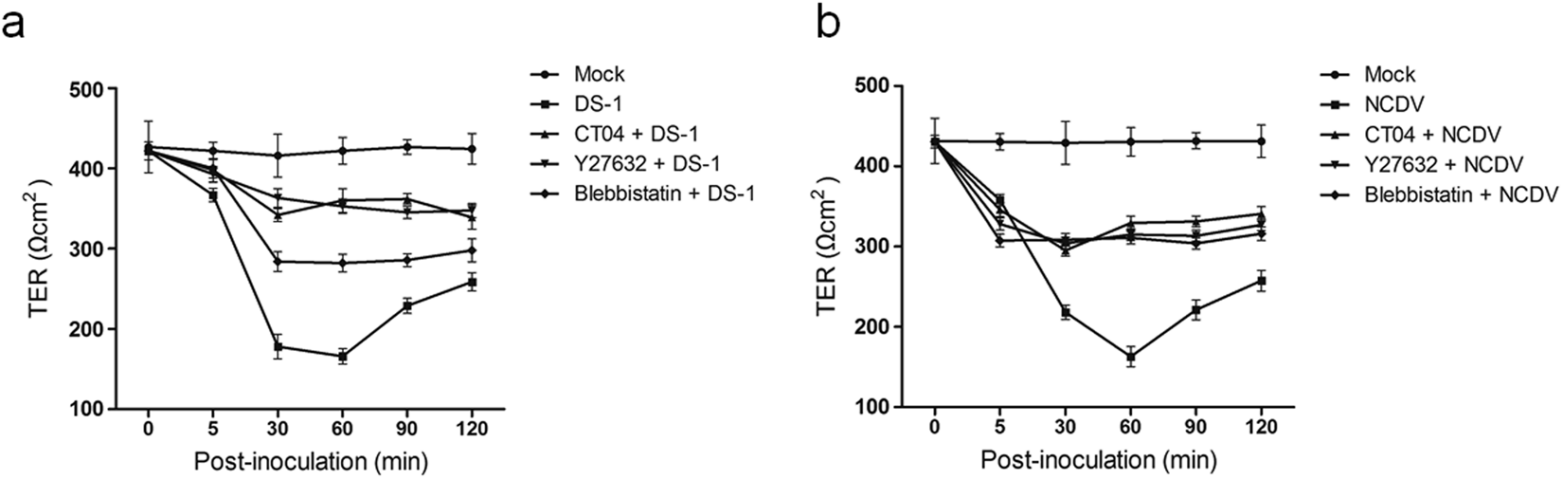
Inhibition of the RhoA/ROCK/MLC pathway restores tight junction resistance in rotavirus-infected polarized epithelial cells. (**a** and **b**) MDCK monolayers grown on transwell filters were pretreated with or without RhoA inhibitor (CT04), ROCK inhibitor (Y27632), or MLC inhibitor (blebbistatin) for 1 h at 37 °C and then mock-inoculated or inoculated with RVA strain (**a**) DS-1 or (**b**) NCDV (MOI = 10), followed by determination of TER at the indicated time points. Net TER was calculated by subtracting the background (membrane filter without cells) and multiplying the resistance (Ω) by the area (0.33 cm^2^) of the filter. All experiments were performed in triplicate. Data are presented as the mean ± standard error of the mean from three independent experiments.

Previous *in vitro* studies have shown that RVAs can disrupt TJ barrier function, resulting in reduced TER in polarized epithelial monolayers^7^. First, we evaluated whether infection with RVA strain DS-1 or NCDV caused a decrease in the TER of polarized MDCK cells early in infection. Confluent polarized MDCK cells grown on polyester transwell membranes were either mock-infected or infected apically with RVA strain DS-1 or NCDV, and TER was measured at the indicated time points. TER measurements across the mock-infected cells remained constant (Fig. 5a,b); however, infection with the RVA strains DS-1 and NCDV resulted in a significant decline in the TER of MDCK monolayers at 30 and 60 mpi (Fig. 5a,b), consistent with previous results^7^. To investigate whether RhoA/ROCK/MLC signaling is involved in the reduction of TER associated with RVA infection, MDCK monolayers were pretreated with RhoA (CT04), ROCK (Y27632), or MLC (blebbistatin) inhibitors for 1 h. RVA strains were then added to the monolayers, and TER was measured at the indicated time points. Inhibition of RhoA, ROCK, or MLC restored the TER of MDCK monolayers infected with RVA strains DS-1 (Fig. 5a) and NCDV (Fig. 5b), suggesting that the RhoA/ROCK/MLC pathway mediates disruption of TJ function induced by early RVA infection.

### Rotavirus-induced early activation of RhoA/ROCK/MLC signaling modifies the distribution of TJ proteins

**Figure 6:**
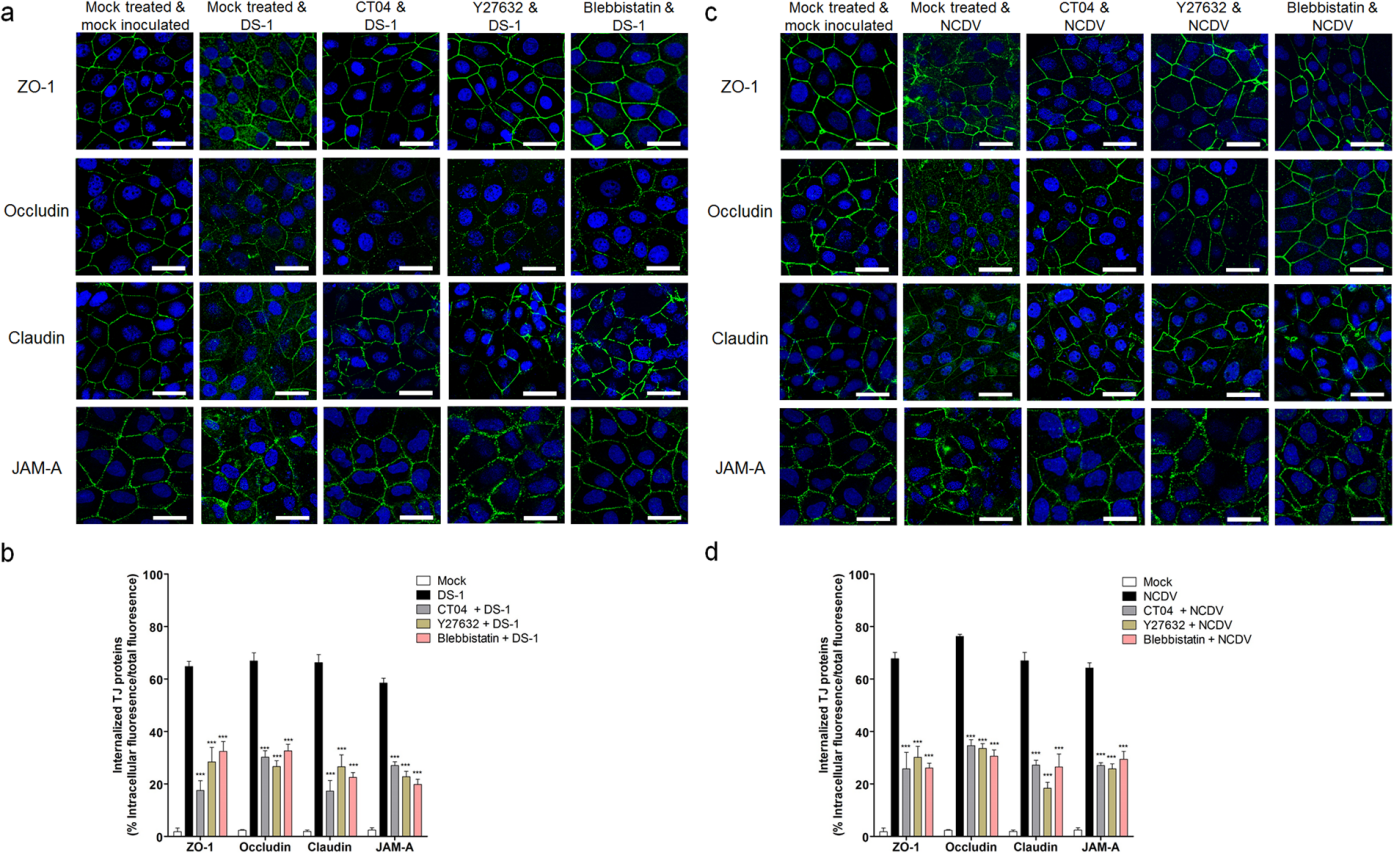
Distribution of tight junction proteins is altered by the rotavirus-induced RhoA/ROCK/MLC signaling pathway. (**a**–**d**) MDCK monolayers were untreated or treated with RhoA inhibitor (CT04), ROCK inhibitor (Y27632), or MLC inhibitor (blebbistatin) for 1 h at 37 °C and then mock-inoculated or inoculated with RVA strain (**a** and **b**) DS-1 or (**c** and **d**) NCDV (MOI = 10) for 1 h. Cells were then fixed, permeabilized, and prepared for confocal microscopy using rabbit anti-ZO-1, occludin, claudin, and JAM-A antibodies and relevant secondary antibodies. All experiments were performed in triplicate and representative images are shown. The scale bars correspond to 20 μm. (**b** and **d**) Quantification of the internalization of each TJ molecule (shown as a percentage of intracellular fluorescence/total fluorescence) as described in Materials and Methods.

Since early RVA infection disrupts TJ proteins in polarized epithelial cells^7^, the effect of the two RVA strains DS-1 and NCDV on the disruption of TJ molecules in polarized MDCK cells was examined. In mock-treated and mock- infected cells, morphological assessment of TJ proteins, occludin, claudin, and JAM-A, as well as the cytosolic scaffold protein ZO-1 revealed smooth arc-like structures along the plasma membrane (Fig. 6). However, during early infection with either RVA strain (DS-1 or NCDV) internalization of these proteins was more pronounced in cells infected with these strains compared with that in control cells (Fig. 6). These results confirm and expand on previous reports, indicating that RVA-infected polarized MDCK cells and non-polarized MA104 cells have an altered distribution of TJ protein and associated scaffold protein^7,32^.

In epithelial cells, the apical actomyosin ring associates with the apical junctional complex and controls its assembly and barrier properties^41–43^. Numerous studies have linked MLC phosphorylation to increased actin polymerization, stress fiber assembly, and *in vivo* and *in vitro* reorganization of TJ proteins^13,44,45^; therefore, we asked whether the changes in the distribution of TJ proteins in RVA-infected cells could be altered by blocking the RhoA/ROCK/MLC pathway. MDCK cells were either mock-treated or treated with RhoA (CT04), ROCK (Y27632), or MLC (blebbistatin) inhibitors, and then infected with RVA strains DS-1 or NCDV. Compared with mock-treated, virus-infected cells, pretreatment of MDCK cells with any of the inhibitors before infection with the RVA strains partially but not completely inhibited the translocation of TJ proteins from the junctional area to the cytoplasm (Fig. 6). These findings indicate that inhibition of RhoA/ROCK/MLC signaling can partially inhibit the intracellular translocation of TJ proteins and the associated scaffold protein ZO-1 from the pericellular plasma junction.

### Inhibition of the RhoA/ROCK/MLC pathways restores the gate and fence functions of TJs tisturbed by rotavirus infection

**Figure 7:**
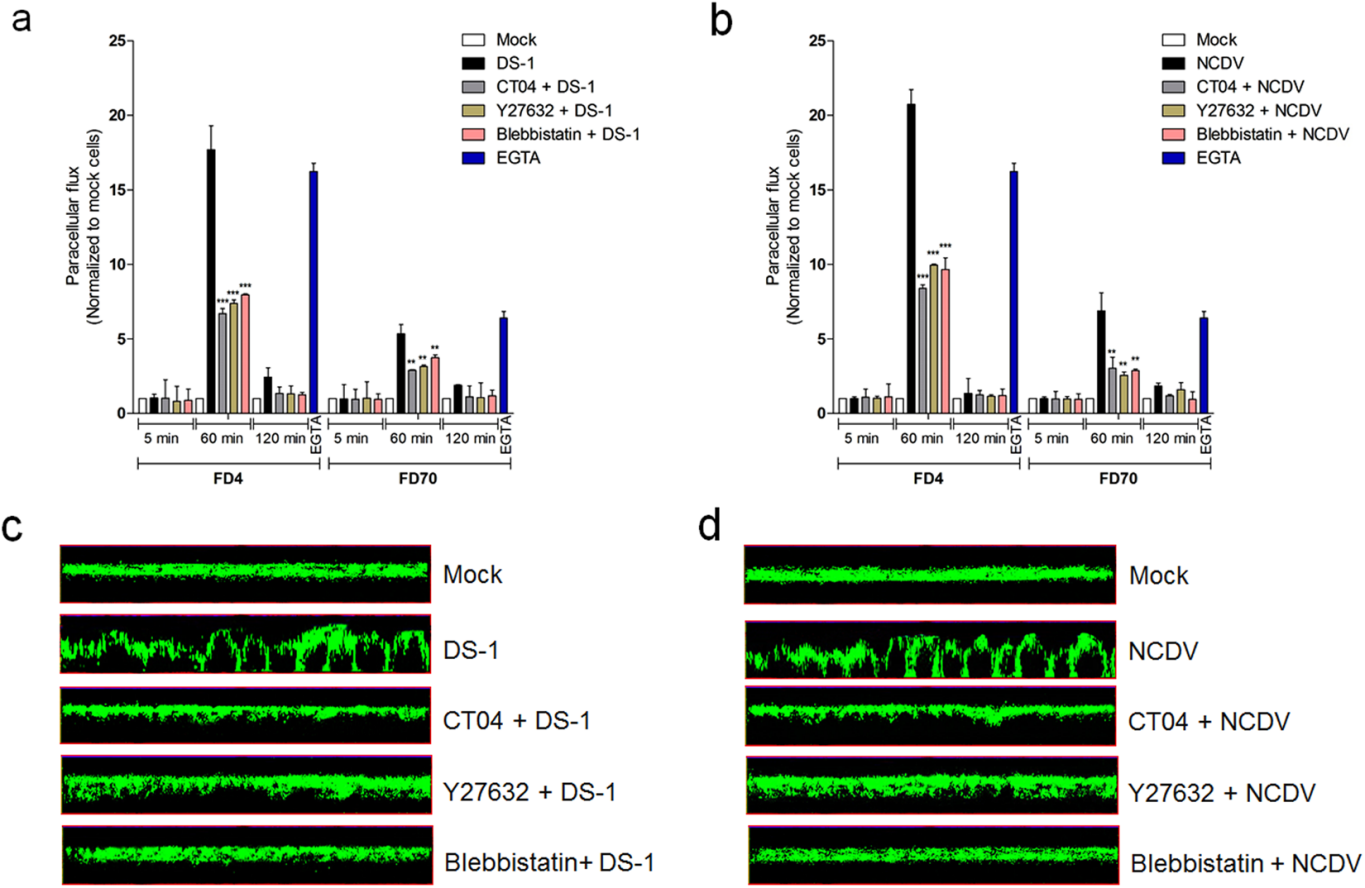
Inhibition of the RhoA/ROCK/MLC pathway restores the gate and fence functions of tight junction in rotavirus-infected polarized epithelial cells. MDCK monolayers grown on transwell filters were untreated or treated with the RhoA inhibitor (CT04), ROCK inhibitor (Y27632), or MLC inhibitor (blebbistatin) for 1 h at 37 °C and then mock-inoculated or inoculated with RVA strain (**a**) DS-1 or (**b**) NCDV (MOI = 10) for 5, 60, or 120 min. (**a** and **b**) Paracellular flux of 4-kDa FITC-dextran (FD4) and 70-kDa FITC dextran (FD70) was measured in the apical to basolateral direction. The amount of FITC dextran diffused to the basolateral side of the monolayer was normalized by the average obtained from control MDCK cells. As a positive control, MDCK monolayers were treated for 10 min with 1.8 mM EGTA. (**c** and **d**) Distribution of the membrane fluorescent marker BodipyFL-C12-sphingomyelin/BSA (5 nmol/ml) loaded onto the apical surface of MDCK cells was determined by z-sectioning using a confocal microscope. All experiments were performed in triplicate; c and d panels show representative results. Data are presented as the mean ± standard error of the mean from three independent experiments. Differences were evaluated using a one-way ANOVA. *p < 0.05; **p < 0.001; ***p < 0.0001.

We examined whether early dissociation of TJs induced by RVA-infection can be restored by inhibition of the associated RhoA/ROCK/MLC signaling pathway. We first examined whether RVA (strains DS-1 and NCDV) altered the gate and fence functions of TJs. TJs restrict the paracellular diffusion of ions and hydrophilic non-ionic tracers in a selective manner, depending on the charge and size of compounds. Paracellular permeability to hydrophilic tracers can be monitored using fluorescently labelled compounds such as dextran. Here, we used 4 kDa and 70 kDa FITC-dextrans (FD4 and FD70, respectively) to evaluate TJ permeability. Consistent with a previous report^7^, the permeability of polarized MDCK cells to FD4 and FD70 was found significantly increased at 60 mpi but not at 5 or 120 mpi after infection with the RVA strains or after incubation with 1.8 mM ethylene glycol-bis(β-aminoethyl ether)-N,N,N′,N′-tetraacetic acid (EGTA) for 10 min, which is known to open TJs and causes epithelial depolarization, compared with that observed in mock-infected cells (Fig. 7a,b). We next examined whether inhibition of the RhoA/ROCK/MLC pathway could restore the gate function of TJs disturbed by early RVA infection. MDCK cells were pretreated for 1 h with RhoA (CT04), ROCK (Y27632), or MLC (blebbistatin) inhibitors and then infected with RVA strain DS-1 or NCDV for 60 min. Pretreatment with each RhoA/ROCK/MLC inhibitor significantly reduced transepithelial leakage of the polarized MDCK cells (Fig. 7a,b).

TJs can also restrict the exchange of membrane molecules, proteins and lipids, between the apical and basolateral domains of the cell membrane, which is termed fence function^6^. The fence function of the TJ can be evaluated in monolayers cultured on transwell membranes by adding a fluorescent lipid (BodipyFL-C12-sphingomyelin-BSA complex) onto the apical membrane and observing whether the fluorescent label reaches the basolateral membrane^7,46,47^. Therefore, mock-pretreated or pretreated MDCK monolayers grown on transwell filters were either mock-infected or infected with RVA strain DS-1 or NDCV for 1 h. The apical compartments were then incubated with BodipyFL-C12-sphingomyelin/BSA complex, and the cell membranes were immediately analyzed by confocal microscopy. Mock-treated and mock-infected MDCK monolayers retained the fluorescent lipid in the apical part of the cell as it did not diffuse towards the lateral regions (Fig. 7c,d). However, in RVA-infected cells, the fluorescent lipid began to diffuse through the TJs and labeled the lateral plasma membrane (Fig. 7c,d), consistent with a previous report^7^. When the cells were pretreated with RhoA (CT04), ROCK (Y27632), or MLC (blebbistatin) inhibitors, lateral diffusion of the fluorescent lipid was visibly reduced (Fig. 7c,d). Taken together, these results suggest that the RVA-induced early disturbances of gate and fence functions of TJs by contraction of the actomyosin ring through RhoA/ROCK/MLC signaling can be restored by inhibition of the same pathway.

## Discussion

RVAs enter cells through a complex multistep process in which different domains of RVA outer proteins (VP8*, VP5*, and VP7) as attachment and entry receptors interact with various cell surface molecules^21,22^. Moreover, entry of RVAs into cells occurs basolaterally through binding to the TJ protein JAM-A as a coreceptor possibly with the help of another TJ protein occludin and its associated scaffold protein, ZO-1^1,32^. For RVAs to bind to JAM-A, it is a prerequisite that the TJ is dissociated. Confirming a previous report that showed how the VP8* protein can open TJs in MDCK cells^7^, infection with RVA strains DS-1 and NCDV and treatment of cells with cogent VP8* proteins led to the dissociation of TJs of polarized MDCK cells at early time points, as evidenced by a decrease in TER and an increase in paracellular flux. Our data further demonstrated that the dissociation of TJs in MDCK cells, as a result of rotavirus infection or VP8* treatment, is due to the activation of the RhoA/ROCK/MLC signaling pathway. Interactions between viral ligands and cellular receptors result in the activation of cell signaling pathways to facilitate entry and optimize infection^48^. Activation of RhoA/ROCK/MLC signaling leads to contraction of the actomyosin ring and subsequent opening of TJs, allowing viral access to the JAM-A coreceptor and entry into the cell^7,21,22,49^.

Cytoskeletal regulation of the TJ barrier is best represented by contraction of the perijunctional actomyosin ring, which is immediately beneath the TJ^4,13,50^. This event appears to be controlled by phosphorylation of the 20-kDa MLC protein^4,13,50^. Contraction of this ring exerts tension on the cell membrane, which is transmitted to the TJ, thus attenuating its barrier function as a result of the numerous interactions of specific TJ proteins, such as ZO-1, occludin, and claudin with actomyosin^4,13,50^. The RhoA/ROCK signaling pathway is linked to structural and physiological dysfunction of TJs and is key for MLC phosphorylation^4,13^. Activation of this signaling pathway or RhoA GTPase has also been observed during the entry, infection, and/or spread of other viruses in polarized and non-polarized cells, including influenza virus^51^, Kaposi sarcoma-associated herpesvirus^52^, Marek’s disease herepesvirus^53^, herpes simplex virus^54^, human immunodeficiency virus^55,56^, hepatitis C virus^57^, measles virus^58^, and respiratory syncytial virus^59^. In this study, MLC phosphorylation detected at an early stage in RVA-infected cells was associated with activation of the upstream effectors, RhoA and ROCK, whose activity was reduced in response to corresponding inhibitors.

RhoA/ROCK-dependent phosphorylation of MLC can be triggered indirectly by inhibiting MLC dephosphorylation by pMYPT, an inhibitor of MLC phosphatase^4,13^. In RVA-infected MDCK cells, pMYPT levels were not found increased by western blot assays nor was it precipitated by IP, suggesting that MLC phosphorylation by pMYPT activation via RhoA/ROCK signaling is not involved in RVA-induced early dissociation of TJs. In contrast, MLC phosphorylation, as observed in the early RVA-infected cells, can induced by the PKC/MLCK signaling pathway^4,13^. Unlike the influenza-induced activation of PKC signaling^37^, the present study showed that in early RVA-infected cells, pPKC levels did not increase and neither RVA-induced pMLC levels were reduced by inhibitors specific for PKC or MLCK. MLCK, a downstream kinase of PKC, is reported to not contribute to TJ disruption during RVA infection, as treatment of Caco-2 cells with the MLCK inhibitor ML-9 did not reduce time of onset or rate of loss of TER^60^. These results suggest that the PKC/MLCK signaling pathway is not associated with the activation of MLC observed in early RVA-infected MDCK cells.

The disruption of TJs observed in this study and in a previous study^7^ due to infection of MDCK cells with RVAs differs from that observed during RVA replication and release. For example, disruption of TJs characterized by a progressive, post-infection time-dependent decrease in TER and an increase in paracellular permeability has been reported to be involved in distribution of TJ-associated proteins^60,61^. Thereafter, disruption of TJs was reported to occur in response to the RVA enterotoxin, NSP4^62^, which is responsible for increased cytosolic Ca^2+^ and diarrhea in mice induced by stimulation of phospholipase C-mediated inositol 1,4,5-trisphosphate production^63–65^. Reduction in sucrase-isomaltase expression in Caco-2 cells during the late phase of infection with RRV strain can be induced by perturbation of protein targeting and organization of the microvillar cytoskeleton^66^, possibly through cAMP/PKA signaling^67^. RRV infection of Caco-2 cells affects the distribution and mRNA levels of occludin via the cAMP/PKA pathway, independent of virus-induced apical F-actin rearrangement in late RVA-infected cells^61,68^. The RVA VP4 spike protein interacts with actin through a treadmilling process for the final assembly and apical release of progeny virions in polarized Caco-2 cells and possibly also for brush border alterations^69,70^. The RVA strains, DS-1 and NCDV, used in this study are known to be late-penetrating viruses due to the uncoating and release of their double-layered particles (occurring at 80–90 and 90–100 mpi for NCDV and DS-1 strains, respectively) in the late endosome^71^. In the present study, the RVA-induced disruption of TJs, characterized by a drop in TER and activation of its associated signaling molecules (RhoA, ROCK, and pMLC), was detected as early as 5 min after treatment of cells with their VP8* proteins as well as after inoculation of cells with RVA strains DS-1 and NCDV. Nevertheless, the RVA-induced late disruption described above differs from that caused by the early RVA infection observed in this study and in a previous report^7^.

Many pathogens use an array of tactics to commandeer and disrupt junctional structures to their advantage. This is achieved through two general arms: alteration and degradation of specific TJ proteins define one arm and cytoskeletal-mediated events define the other^2^. Consistent with previous reports^1,7^, infection of polarized MDCK by RVA strains DS-1 and NCDV or treatment with their VP8* proteins, induced translocation of TJ proteins and the scaffold protein ZO-1 into the cytoplasm. Chemical inhibitors specific for each of the signaling molecules (RhoA, ROCK, and MLC) partially restored RVA-induced intracellular translocation of TJ proteins to the perijunctional region and somewhat abrogated the virus-induced decrease in TER and increase in paracellular flux.

Since activation of the RhoA/ROCK pathway induces barrier dysfunction and increases permeability in various diseases^1,6,8–11^, there have been attempts to therapeutically inhibit the RhoA/ROCK pathway^37,72,73^. RVA-induced early dissociation of TJs was restored by the RhoA inhibitor CT04, the ROCK inhibitor Y27632, and the MLC inhibitor blebbistatin. Among therapeutic candidates aimed at inhibiting the RhoA/ROCK pathway, fasudil hydrochloride (HA-1077), a derivative of isoquinoline and the only clinically approved pharmacological inhibitor of ROCK, has been used for the treatment of cerebral vasospasm since 1995^37,72–74^. In addition, treatment with fasudil is effective in improving lipopolysaccharide (LPS)-induced endothelial barrier dysfunction and LPS-induced production of pro-inflammatory cytokines^75–78^. Therefore, therapeutic approaches aimed at enhancing or restoring intestinal epithelial barrier function can potentially contribute to adjunct therapy for the reversal of RVA-induced early disruption as well as RVA diarrhea.

In summary, our findings demonstrate that early activation of RhoA/ROCK/MLC in polarized MDCK cells by RVAs or its VP8* proteins leads to redistribution of TJ proteins and their scaffold protein into the cytoplasm, causing reduced TER and increased paracellular permeability. Since disruption of TJs can be induced by the interaction of various signaling pathways and molecules^2,4,6,12^, future studies should address the possibility that other mechanisms may contribute to the RVA-induced early dissociation of TJs.

## Materials and Methods

### Cells and viruses

Madin-Darby canine kidney (MDCK) cells from the American Type Culture Collection (ATCC, Manassas, VA, USA) were grown in Dulbecco’s modified Eagle’s medium (DMEM; Welgene, Daegu, South Korea) supplemented with 10% fetal bovine serum (FBS), 100 U/ml penicillin, and 100 µg/ml streptomycin. Monkey kidney MA104 cells (ATCC) were grown in alpha minimal essential medium (Welgene) supplemented with 10% FBS, 100 U/ml penicillin, and 100 μg/ml streptomycin.

The human rotavirus DS-1 (G2P1B[4]) and bovine rotavirus NCDV (G6P6[1]) strains purchased from ATCC were preactivated with 10 μg/ml crystalized trypsin (Cat. No. 27250-018; Gibco, Fort Worth, TX, USA) and propagated in MA104 cells as described previously^71^. Each viral titer was determined by cell culture immunofluorescence (IF) assay using monoclonal antibodies (Mabs) specific for RVA VP6 protein and was expressed as fluorescence focus units per milliliter (FFU/ml).

### Reagents and antibodies

RhoA inhibitor (CT04) was purchased from Cytoskeleton, Inc. (Denver, CO, USA), and ROCK inhibitor (Y27632), MLC inhibitor (blebbistatin), MLCK inhibitor (ML7), and PKC inhibitor (Gö 6983) were from Sigma-Aldrich (St. Louis, MO, USA). EGTA was purchased from Santa Cruz Biotechnology (Dallas, Texas, USA). SlowFade Gold antifade reagent with 4′,6-diamidino-2-phenylindole (DAPI) was obtained from Molecular Probes (Bedford, MA, USA). Specific rabbit polyclonal antibodies against pMLC (Ser19), PKCα, MYPT1, and pMYPT1 (at Thr853) as well as rabbit Mabs against MLC2, pan phosphorylated PKC (pPKC), and ROCK were purchased from Cell Signaling Technology (Beverly, MA, USA). Rabbit anti-MLCK Mab was obtained from Abcam (Cambridge, MA, USA). Goat anti-occludin polyclonal antibody was purchased from Santa Cruz Biotechnology, rabbit anti-claudin and JAM polyclonal antibodies were obtained from Invitrogen (Carlsbad, CA, USA), and rabbit anti-ZO-1 polyclonal antibody was purchased from Life Technologies (Carlsbad, CA, USA). Rabbit anti-glyceraldehyde-3-phosphate dehydrogenase (GAPDH; FL-335) polyclonal antibody was purchased from Santa Cruz Biotechnology. Mouse anti-RVA VP6 was purchased from Median Diagnostic (Chuncheon, South Korea). Secondary antibodies included horseradish peroxidase (HRP)-conjugated goat anti-rabbit IgG (Cell Signaling Technology), HRP-conjugated goat anti-mouse IgG (Ab Frontier, Seoul, South Korea), and AF488-conjugated goat anti-rabbit IgG (Life Technologies).

### Cytotoxicity assay

Gö6983 and blebbistatin were dissolved in dimethyl sulfoxide (DMSO), while CT04, Y27632 and ML7 were dissolved in distilled water (DDW) to prepare stock solutions. The cytotoxic effects of the chemicals and their solvents were tested, using the 3-(4,5-dimethylthiazol-2-yl)-2,5-diphenyl tetrazolium bromide (MTT) assay as described previously^79^. Briefly, MDCK cells gown in 96-well plates were incubated in media containing different concentrations of various chemicals for 24 h. After removing the media, 200 μl of MTT solution was added to each well and incubated for 4 h at 37 °C in a CO_2_ incubator. Afterwards, 150 μl of DMSO was added to each well and samples were incubated for 10 min at room temperature. The absorbance was measured using an enzyme-linked immunosorbent assay (ELISA) reader at an optical density (OD) of 570 nm. The percent cell viability was calculated using the following formula, [(OD_sample_ − OD_blank_)/(OD_control_ − OD_blank_)] × 100. All chemicals were used at concentrations that did not promote any changes in cell viability. In each experiment, chemicals were freshly diluted to the desired concentration with serum free media before being added to cell monolayers.

### Cloning, expression, and purification of RVA VP8* proteins

Recombinant VP8* proteins from RVA strains DS-1 and NCDV were cloned, expressed, and purified as described previously^71^. Briefly, cDNA encoding the VP8* domain of the DS-1 and NCDV strains was cloned into the expression vector pGEX-4T-1 (glutathione S-transferase [GST]-gene fusion system; GE Healthcare Life Sciences, Piscataway, NJ, USA). After sequence confirmation, recombinant GST-VP8* fusion proteins were expressed in *Escherichia coli* strain BL21. Expression of each domain was induced using isopropyl-b-D-thiogalactopyranoside (IPTG; 0.2 mM) at room temperature overnight. RVA GST-VP8* fusion proteins were purified using the Pierce GST Spin Purification Kit (Waltham, MA, USA) according to the manufacturer’s protocol. Concentration of the purified RVA VP8* domain was determined by measuring the absorbance at 280 nm.

### Treatment of cells with chemical inhibitors

Confluent MDCK cells grown in 6 well plates or 8-well chamber slides for 5–6 days were washed twice with phosphate-buffered saline (PBS, pH 7.4). Cells were either mock-pretreated or pretreated with inhibitory chemicals for 1 h at 37 °C at the following concentrations: CT04 (20 ng/ml and 2 μg/ml), Y27632 (0.1 μM and 20 μM), blebbistatin (10 μM), Gö 6983 (10 nM and 1 μM), and ML7 (0.1 μM and 10 μM). Afterwards, the cells were washed twice with PBS, incubated with RVA strains for the indicated time, and then used for assessment of signaling pathways and measurement of virus titer by IF assay, genome copy number by RT-qPCR, and protein expression by western blotting as described below.

### Western blot analysis

Confluent MDCK monolayers grown in 6 or 12-well plates for 5–6 days were pretreated with or without various inhibitors, mock-infected or infected with RVA strains, or incubated with or without recombinant RVA VP8* proteins (10 μg/ml) for the indicated periods of time. Cells were then washed three times with cold PBS and lysed using cell extraction buffer containing 10 mM Tris/HCl pH 7.4, 100 mM NaCl, 1 mM EDTA, 1 mM EGTA, 1 mM NaF, 20 mM Na_2_P_2_O_7_, 2 mM Na_3_VO_4_, 1% Triton X-100, 10% glycerol, 0.1% sodium dodecyl sulfate (SDS), and 0.5% deoxycholate (Invitrogen) supplemented with protease and phosphatase inhibitors (Roche, Basel, Switzerland) for 30 min on ice. Cell lysates were collected by centrifugation at 12,000 × g for 10 min at 4 °C. Supernatants were analyzed for total protein content using a BCA protein assay kit (ThermoFisher Scientific). Samples were resolved by SDS-polyacrylamide gel electrophoresis (SDS-PAGE) and transferred onto nitrocellulose membranes (GE Healthcare Life Sciences). Membranes were blocked for 1 h at room temperature using Tris-buffered saline containing 5% skim milk before being incubated overnight at 4 °C with the indicated primary antibodies. Bound antibodies were then incubated with HRP-labeled secondary antibodies, after which immunoreactive bands were developed by enhanced chemiluminescence (ECL; Dogen, Seoul, South Korea) and detected using a Davinch-K Imaging System (Youngwha Scientific Co., Ltd, Seoul, South Korea).

### RhoA activation assay

RhoA activation was determined using a RhoA activation assay kit (Cell Biolabs, Inc., San Diego, CA, USA) according to the manufacturer’s recommendations. Briefly, MDCK monolayers were mock-infected, infected with DS-1 or NCDV strains (MOI = 10), or incubated with recombinant RVA VP8* proteins (10 μg/ml). At the indicated time points, cells were washed twice with ice-cold PBS (pH 7.2) and lysed in ice-cold 1X lysis buffer (125 mM HEPES pH 7.5, 750 mM NaCl, 5% NP-40, 50 mM MgCl_2_, 5 mM EDTA, and 10% glycerol) containing protease inhibitor (Roche). Cells were detached by scraping with a cell scraper and the lysates were clarified by centrifugation for 10 min (14,000 × g at 4 °C). Supernatants were incubated with Rhotekin RBD agarose beads for 1 h at 4 °C to pull down RhoA. Next, beads were pelleted by centrifugation for 10 sec at 14,000 × g at 4 °C and washed three times with 1X assay buffer. After the final wash, the beads were resuspended in 2X SDS-PAGE sample buffer, boiled for 5 min, and centrifuged again, and the supernatants were subjected to 12% SDS-PAGE, followed by western blot analysis. Separated proteins were immunoblotted using mouse anti-RhoA Mab.

### Immunofluorescence and confocal microscopy

Polarized MDCK cells grown in 8-well chamber slides pretreated with or without inhibitory chemicals were infected with or without RVA strains DS-1 or NCDV (MOI = 10) or pretreated with or without VP8* proteins (10 μg/ml) for the indicated times. Virus inocula or VP8* proteins were removed, and the cells were washed twice with PBS, fixed with −20 °C methanol for 5 min, and permeabilized by the addition of 0.2% Triton X-100 for 5 min on ice before being washed with PBS. The chamber slides were then incubated with primary antibodies against each TJ protein (1:100 dilution) at 4 °C overnight. Subsequently, cells were washed three times with PBS and incubated with AF488-conjugated goat anti-rabbit secondary antibody (1:100 dilution) for 1 h at room temperature. Cells were then washed with PBS containing 0.1% new born calf serum (PBS-NCS) and mounted with SlowFade Gold antifade reagent containing 1X DAPI solution for nuclei staining. Cells were observed with an LSM 510 confocal microscope and analyzed using LSM software (Carl Zeiss; Jena, Germany). Quantification of TJ molecule internalization was determined by measuring the relative fluorescence intensity profiles (in arbitrary units) of each TJ molecule in the cytoplasm as described previously^80^. Briefly, 10 images of cells treated with the conditions described above were taken by confocal microscopy and then processed and quantified using the ImageJ program (http://rsb.info.nih.gov/ij/). Internalization was calculated by the following equation, internalization = (F_C_)/(F_TOTAL_) × 100%, where F_C_ is the relative fluorescence of each TJ molecule in the cytoplasm and F_TOTAL_ is the total TJ fluorescence. Calculations were based on approximately 50 cells. Internalization of each TJ molecule was expressed as a percentage of mock-treated, mock-infected control.

To determine the RVA infectivity titer, an IF assay was performed as described previously^71^. Briefly, MDCK cells grown in 12-well plates pretreated with or without chemicals were independently infected with the trypsin-pre-activated strains DS-1 and NCDV (10 μg/ml crystalized trypsin) for 12 h. After three freeze-thaw cycles, a 10-fold dilution of each sample was used to infect confluent MA104 cells grown in 96-well plates in triplicate. After an adsorption period of 1 h at 37 °C, virus inocula were removed and the cells were washed with PBS. Subsequently, the infection was allowed to continue at 37 °C for 16 h in media containing 1 μg/ml crystalized trypsin. Cells were then fixed with 80% cold acetone. After washing with PBS (pH 7.4), each well was incubated with Mab against the RVA VP6 protein at 4 °C overnight. Afterwards, the cells were washed three times with PBS, and FITC-conjugated secondary antibodies were added. After washing with PBS (pH 8.0), the nuclei were stained with DAPI. Viral titers are expressed as FFU/ml.

### Real-time RT-PCR

To quantify the total viral RNA of RVA, real-time RT-PCR was carried out as described previously^71^. MDCK cells grown in 12-well plates were pretreated with or without the indicated concentration of chemicals. Cells were then infected with the DS-1 or NCDV strains at a MOI = 10 for 1 h. Next, unbound viruses were removed by washing the cells with PBS. At 12 h post-infection, the cell cultures were washed twice with PBS, harvested by freezing and thawing three times, and cell debris was collected by centrifugation at 2,469 × g for 10 min at 4 °C. Supernatants and remaining bulk samples were collected and stored at −80 °C until used. Total RNA was extracted using an RNeasy kit (Qiagen) according to manufacturer’s instructions. Viral genome copy numbers were determined by one-step SYBR Green real-time RT-PCR, using a primer pair specific for the RVA VP6 gene^71^. Each reaction mixture had a total volume of 20 μl containing 4 μl RNA template (1 μg), 10 μl SensiFast SYBR Lo-ROX One step mixture (Bioline, Quantace, London, UK), 0.8 μl each of forward and reverse primers (10 pmol), 0.2 μl reverse transcriptase, 0.4 μl RiboSafe RNase inhibitor, and 3.8 μl RNase-free water. Real-time RT-PCR was performed using a Rotor-Gene Real-Time Amplification System (Corbett Research, Mortlake, Australia) with the following conditions, reverse transcription was carried out at 50 °C for 30 min, followed by activation of hot-start DNA polymerase at 95 °C for 10 min and 40 cycles of 95 °C for 15 s, 50 °C for 30 s, and 72 °C for 20 s. Quantitation of viral RNA was carried out using a standard curve derived from 10-fold serial dilutions of complementary RNA (cRNA) generated by reverse transcription of *in vitro* transcribed control RNA (RVA VP6 gene). The threshold was automatically defined as the initial exponential phase, reflecting the highest amplification rate. A direct relationship between cycle number and the log concentration of RNA molecules initially present in the RT-qPCR reaction was used to calibrate the crossing points resulting from the amplification curves of the samples.

### Immunoprecipitation assay

Immunoprecipitation of target proteins was performed as previously described^71^. Briefly, MDCK grown in 6-well plates were mock-infected or independently infected with DS-1 and NCDV strains at a MOI of 10 FFU/cell and incubated for the indicated time points at 37 °C. Subsequently, cells were washed and lysed as described above. Cell lysates were pre-cleared by incubation with protein A- or G-agarose beads for 30 min at 4 °C. Subsequently, the pre-cleared cell lysates were incubated with antibodies against ROCK or pMYPT overnight at 4 °C. Immune complexes were captured by incubation with protein A- or G- agarose beads for 1 h at 4 °C, and then immunoprecipitated proteins were evaluated by western blot analysis as described above.

### TER measurements

The integrity and degree of TJ sealing were assessed by measuring the TER as described previously^60–62,81^. Polarized MDCK cells grown as monolayers on polyester membrane transwells (Cat. No. 3470; Corning-Costar, NY, USA) were treated with or without chemicals. After mock-infection or infection with RVA strains DS-1 or NCDV (MOI = 10), the TER was measured using a Millicell ERS-2 epithelial Volt-Ohm Meter (Millipore, Bedford, MA, USA). Electrical resistance in the absence of cells was used as background. Net TER values, presented as ohms × cm^2^ (Ωcm^2^), were calculated by subtracting the background and multiplying the resistance by the surface area of the filter.

### Paracellular flux assay

The permeability of polarized MDCK monolayers was determined by measuring the transepithelial passage of fluorescein-isothiocyanate (FITC) dextran with a molecular mass of 4 and 70 kDa (FD4 and FD70, respectively; Sigma-Aldrich), as described previously^7,60–62^. Briefly, polarized MDCK cells grown on polyester membrane transwells were treated with or without chemicals or infected with or without RVA strains DS-1 or NCDV (MOI = 10) for 5, 60, or 120 min at 37 °C. Another set of monolayers was incubated with 1.8 mM EGTA for 10 min as a positive control. Afterwards, FD4 and FD70 (10 μg/ml) were added to the apical chamber of the transwell, and the cells were incubated for 1 h at 37 °C. After the incubation period, media were collected from the apical and basolateral sides, and the concentration of FITC dextran was determined by a fluorometer (Fluro Max 2; Horiba, Kyoto, Japan) at an excitation wavelength of 492 nm and emission wavelength of 520 nm.

### Membrane lipid diffusion assay

Labelling of cells with fluorescent lipids was performed as previously described^7,46,47^. Briefly, polarized MDCK cells grown on polyester membrane transwell inserts were treated with or without chemicals as well as infected with or without RVA strain DS-1 or NCDV (MOI = 10). Afterwards, cells were labeled on the apical side with 5 nmol/ml solution of the BodipyFL-C12-sphingomyelin-BSA complex (Molecular Probes) for 10 min on ice. The BodipyFL-C12-sphingomyelin-BSA complex was prepared in P buffer (145 mM NaCl, 10 mM HEPES pH 7.4, 1 mM Na-pyruvate, 10 mM glucose, and 3 mM CaCl_2_). Cells were then washed three times with cold P buffer and incubated on ice for 30 min. Next, filters were cut from the frame and mounted in P buffer on glass slides and covered with cover slips. Samples were then analyzed immediately by z- sectioning with an LSM 510 confocal microscope and LSM software.

### Statistical analyses and software

Statistical analyses were performed using data from triplicate experiments with GraphPad Prism version 5.03 (GraphPad Software Inc., La Jolla, CA, USA) and a one-way ANOVA test. *P* values < 0.05 were considered statistically significant. Figures were generated using Adobe Photoshop CS3 and Prism.

## Electronic supplementary material

Supplementary information
