# Supplementary material for: Rotavirus-Induced Early Activation of the RhoA/ROCK/MLC Signaling Pathway Mediates the Disruption of Tight Junctions in Polarized MDCK Cells
Source: Sci Rep. 2018 Sep 17;8:13931. doi: 10.1038/s41598-018-32352-y (PMC6141481; doi:10.1038/s41598-018-32352-y)
Supplement: Supplementary file 1 — Supplementary information [file 41598_2018_32352_MOESM1_ESM.pdf]

Supplementary Information

Rotavirus-Induced Early Activation of the RhoA/ROCK/MLC Signaling Pathway Mediates the Disruption of Tight Junctions in Polarized MDCK Cells

Mahmoud Soliman[1], Eun-Hyo Cho[1], Jun-Gyu Park[1], Ji-Yun Kim[1], Mia Madel Alfajaro[1], Yeong-Bin Baek[1], Deok-Song Kim[1], Mun-Il Kang[1], Sang-Ik Park[1], Kyoung-Oh Cho[1]

[1]Laboratory of Veterinary Pathology, College of Veterinary Medicine, Chonnam National University, Gwangju, Republic of Korea. Correspondence and request for materials should be addressed to Sang-Ik Park (sipark@chonnam.ac.kr), or Kyoung-Oh Cho (choko@chonnam.ac.kr).

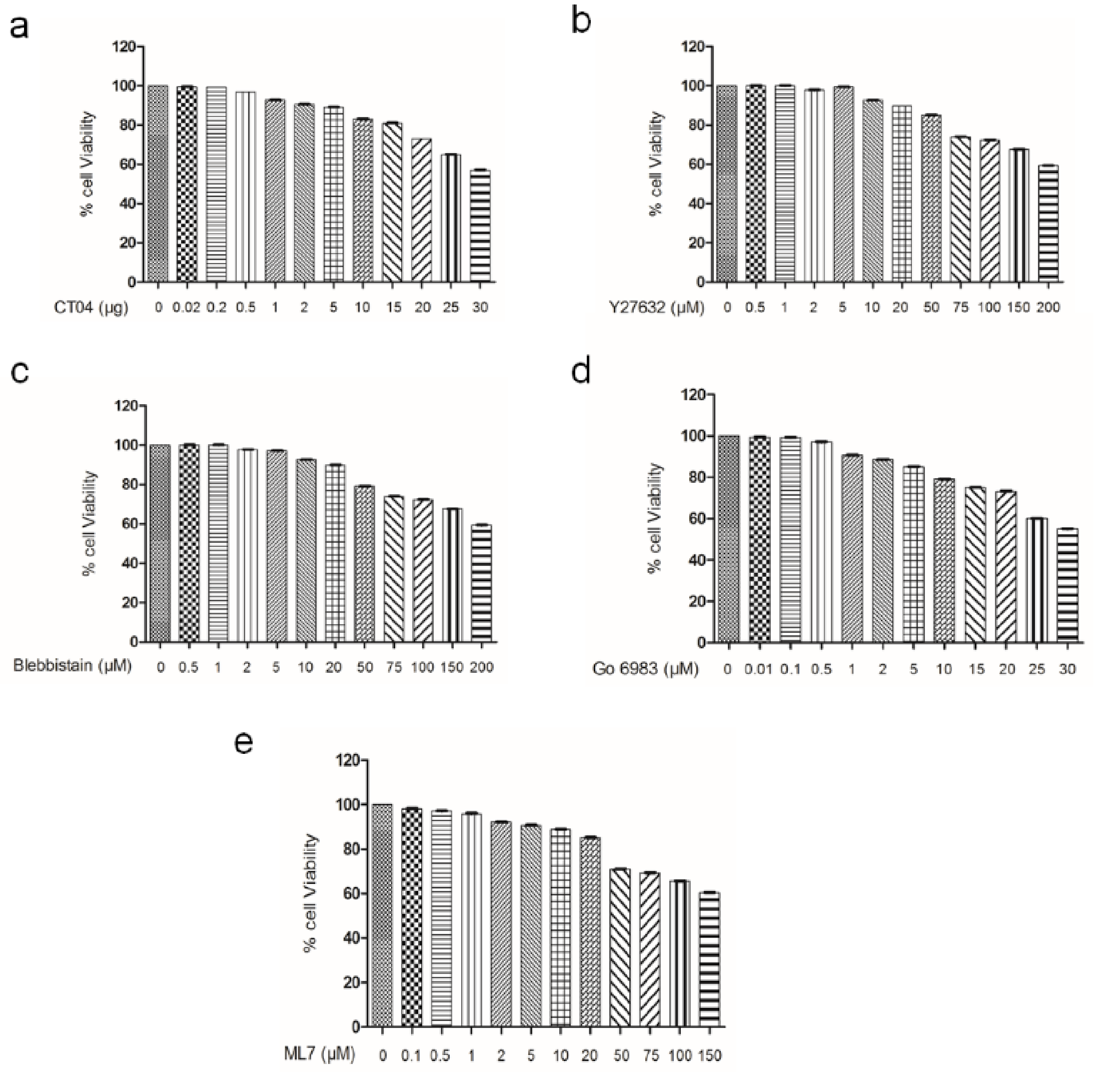

Supplementary Figure S1. Determination of the cytotoxicity of chemicals by MTT assay. (a-e) MDCK cells grown in 96-well plates were incubated with various concentrations of the indicated chemicals for 24 h at 37°C in triplicate. Afterwards, the chemical-containing media was completely removed and replaced with 200 µl of MTT solution for 4 h at 37°C. Each well was incubated with 100 µl DMSO for 10 min at room temperature. Cell viability was measured using an ELISA reader at an OD value of 570 nm.

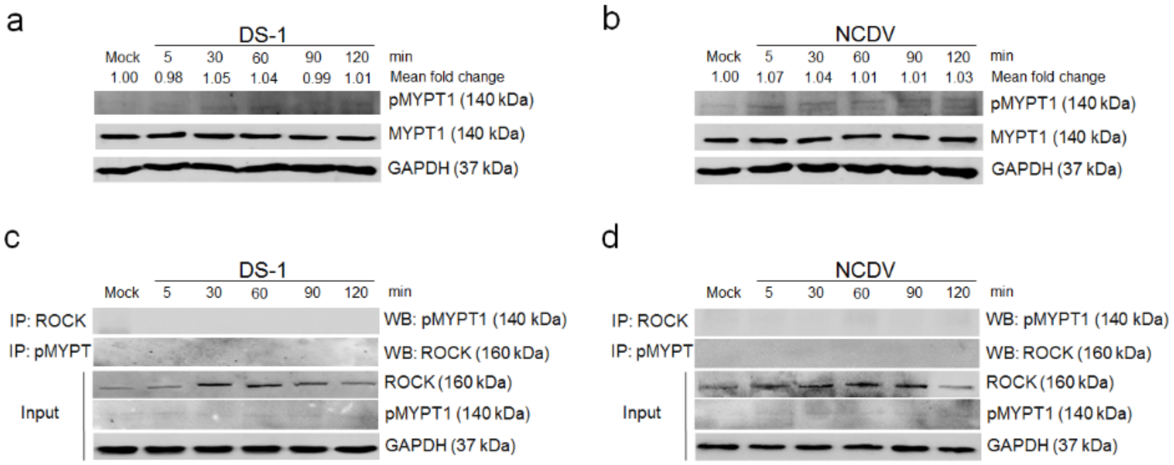

Supplementary Figure S2. Myosin light chain phosphatase (MYPT1) is not activated in MDCK cells during early time of human DS-1 and bovine NCDV strains infection. (a and b) RVA human DS-1 and bovine RVA NCDV strains (MOI = 10) were inoculated into confluent MDCK monolayers. Cells were then harvested at the indicated time points. The cell lysates were subjected to Western blot to determine the expression levels of phosphorylated MYPT1 (pMYPT1) and MYPT1 using the relevant antibodies. GAPDH was used as a loading control. The intensity of pMYPT1 relative to GAPDH was determined by densitometric analysis and indicated above each lane. (c and d) Lysates of MDCK cells either mock-infected or infected with the RVA strains DS-1 or NCDV at the indicated time points were immunoprecipitated using antibodies specific for ROCK or pMYPT. Co-immunoprecipitated proteins were analyzed by Western blot to detect pMYPT or ROCK using the relevant antibody. All experiments were performed in triplicate and representative images of different gels from each group are presented.

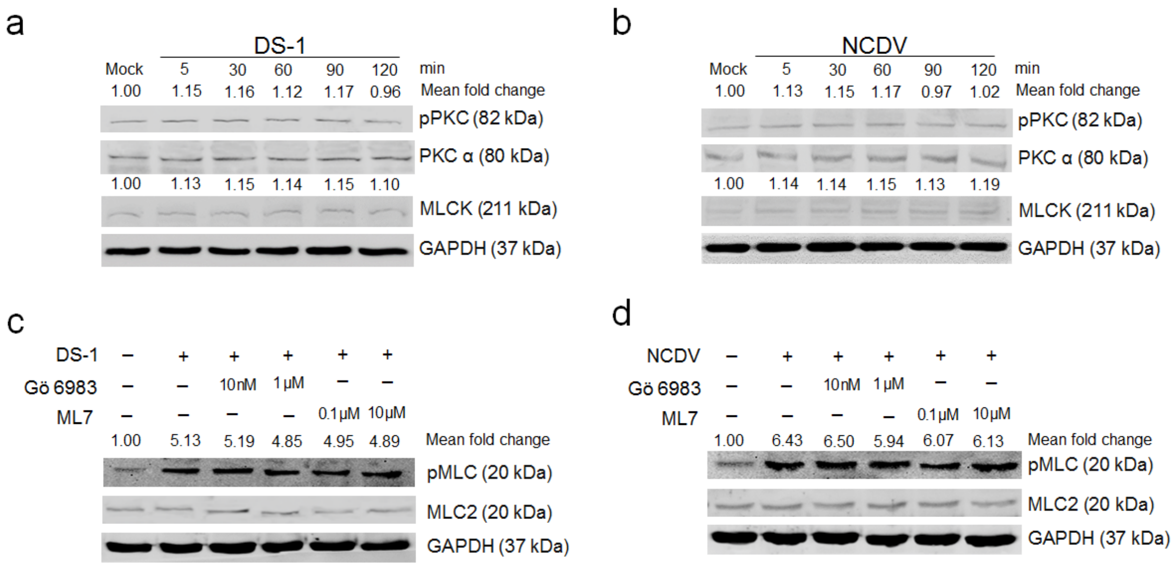

Supplementary Figure S3. Rotavirus-activation of pMLC is independent of PKC/MLCK signaling pathway. (a and b) Confluent MDCK monolayers were either mock-infected or infected with the RVA strains DS-1 (a) or NCDV (b) (MOI = 10), for the indicated time points. Cell lysates were subjected to Western blot to detect of pPKC, PKCα, and MLCK using the relevant antibodies. GAPDH was used as a loading control. (c and d) Confluent MDCK monolayers were either mock-treated or pretreated with the PKC inhibitor Gö 6983 or MLCK inhibitor ML7 for 1 h at 37°C and then infected with RVA strains DS-1 or NCDV (MOI = 10). Cell lysates were harvested after 1 h and the expression level of pMLC evaluated by Western blot analysis. GAPDH was used as a loading control. All experiments were performed in triplicate and representative images of different gels from each group are presented. The intensities of pPKC, MLCK, and pMLC relative to GAPDH were determined by densitometric analysis and indicated above each lane.

Uncropped gel images

Figure 1.

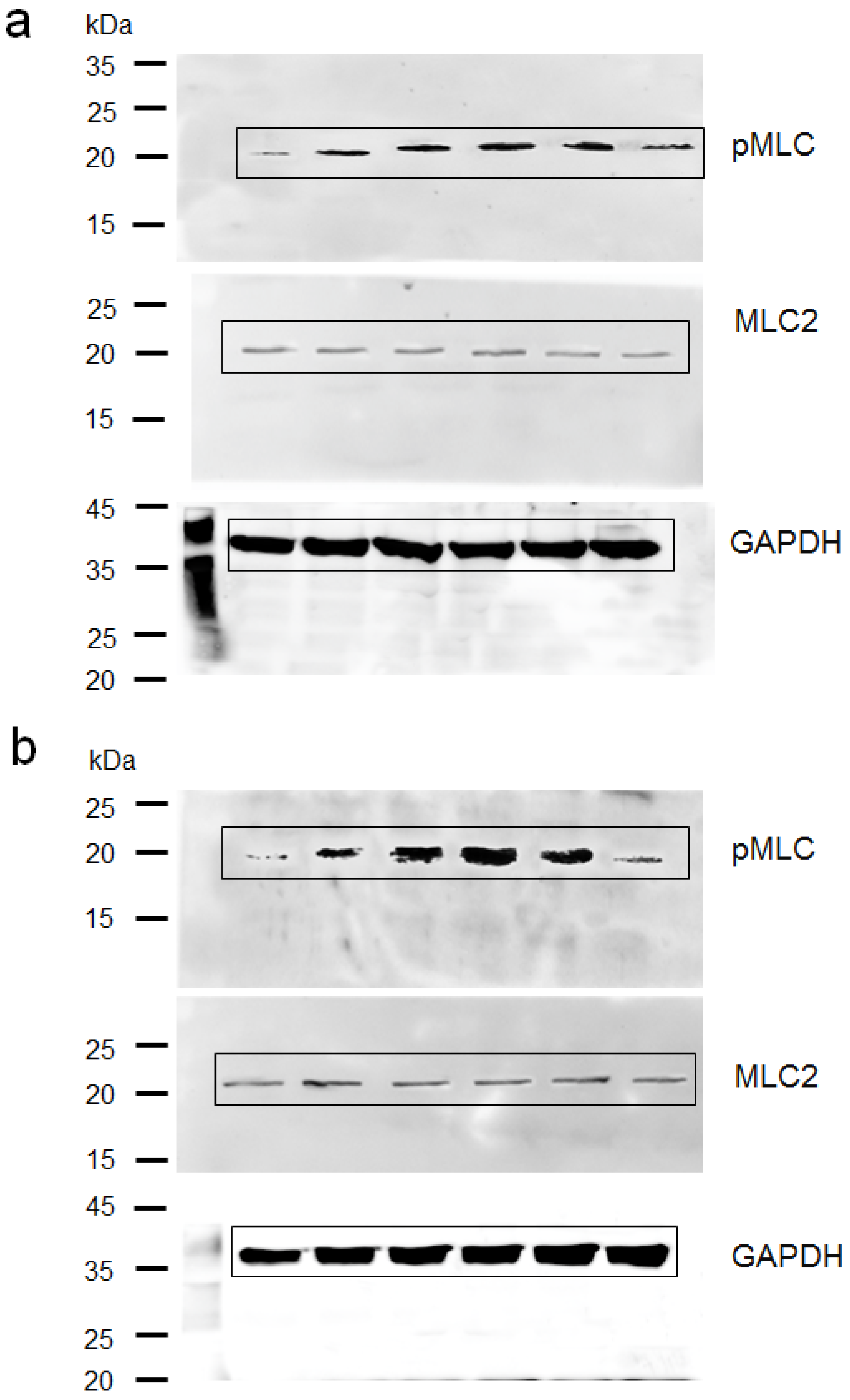

Figure 2.

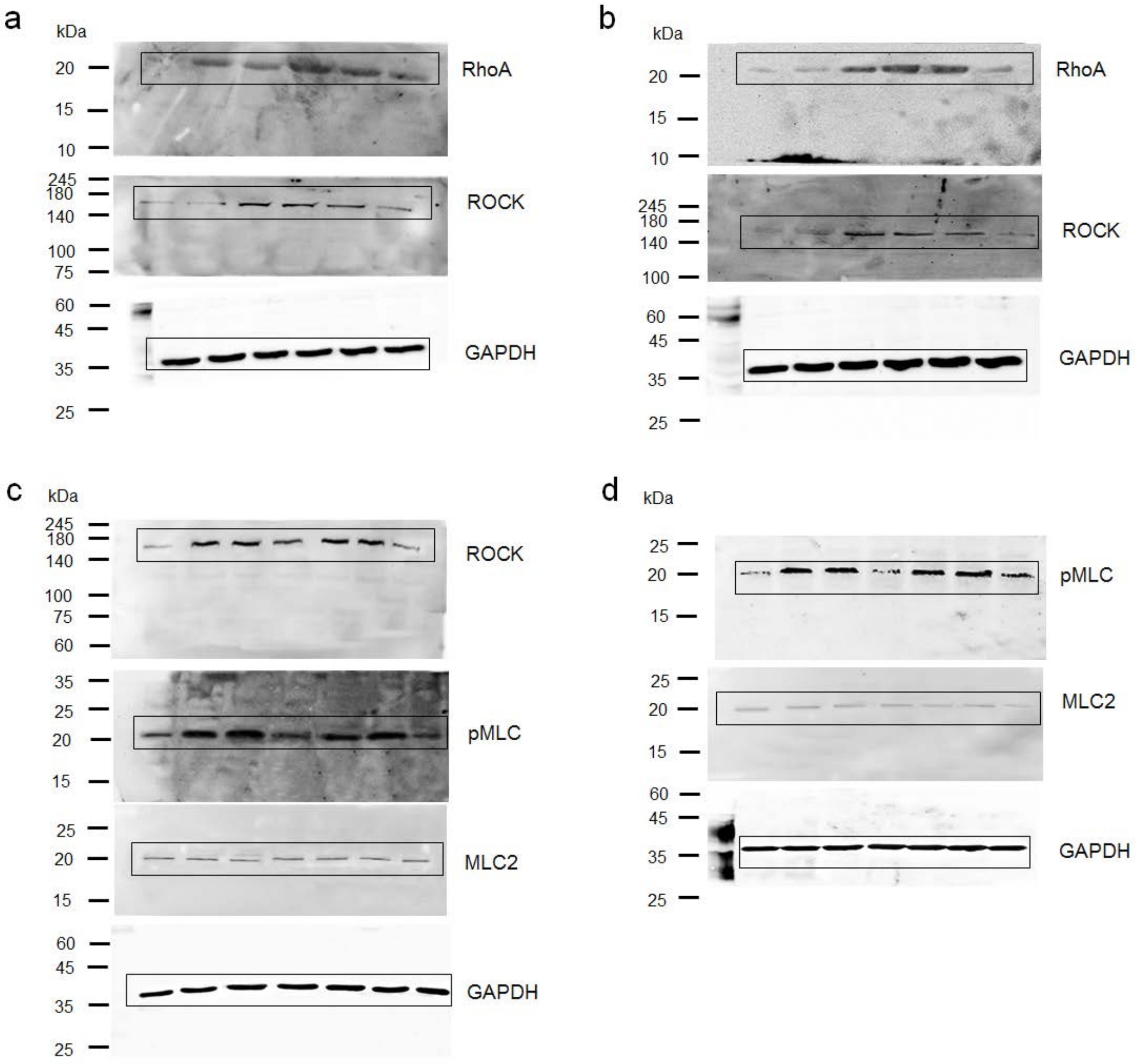
a
kDa
20
15
10
RhoA
245
180
140
100
75
ROCK
60
45
35
25
GAPDH
b
kDa
20
15
10
RhoA
245
180
140
100
ROCK
60
45
35
25
GAPDH
c
kDa
245
180
140
100
75
60
ROCK
35
25
20
15
pMLC
25
20
15
MLC2
60
45
35
25
GAPDH
d
kDa
25
20
15
pMLC
25
20
15
MLC2
60
45
35
25
GAPDH

Figure 3.

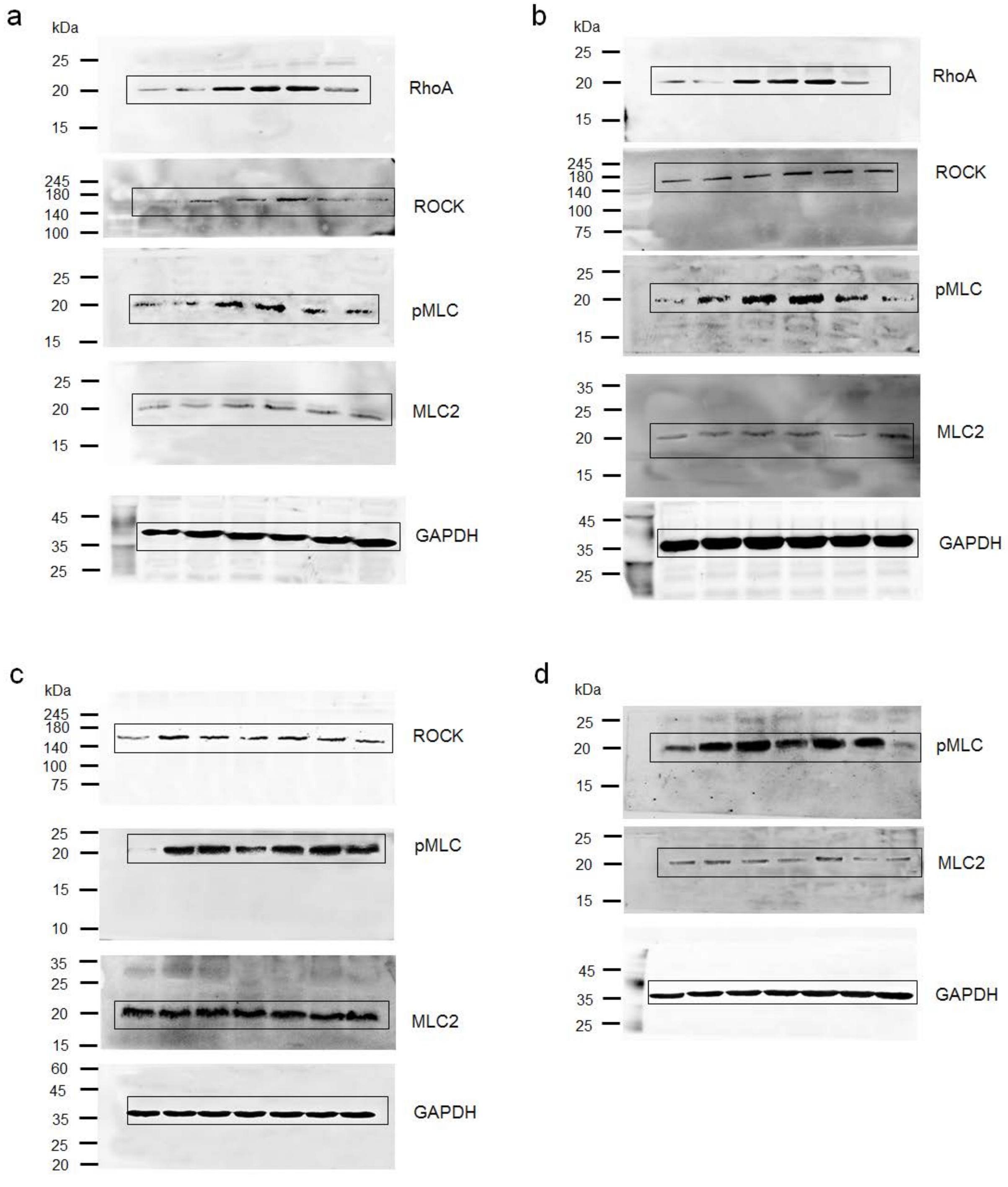
a
kDa
25
20
15
RhoA
245
180
140
100
ROCK
25
20
15
pMLC
25
20
15
MLC2
45
35
25
GAPDH
b
kDa
25
20
15
RhoA
245
180
140
100
75
ROCK
25
20
15
pMLC
35
25
20
15
MLC2
45
35
25
GAPDH
c
kDa
245
180
140
100
75
ROCK
25
20
15
10
pMLC
35
25
20
15
MLC2
60
45
35
25
20
GAPDH
d
kDa
25
20
15
pMLC
25
20
15
MLC2
45
35
25
GAPDH

Figure 4 c and d.

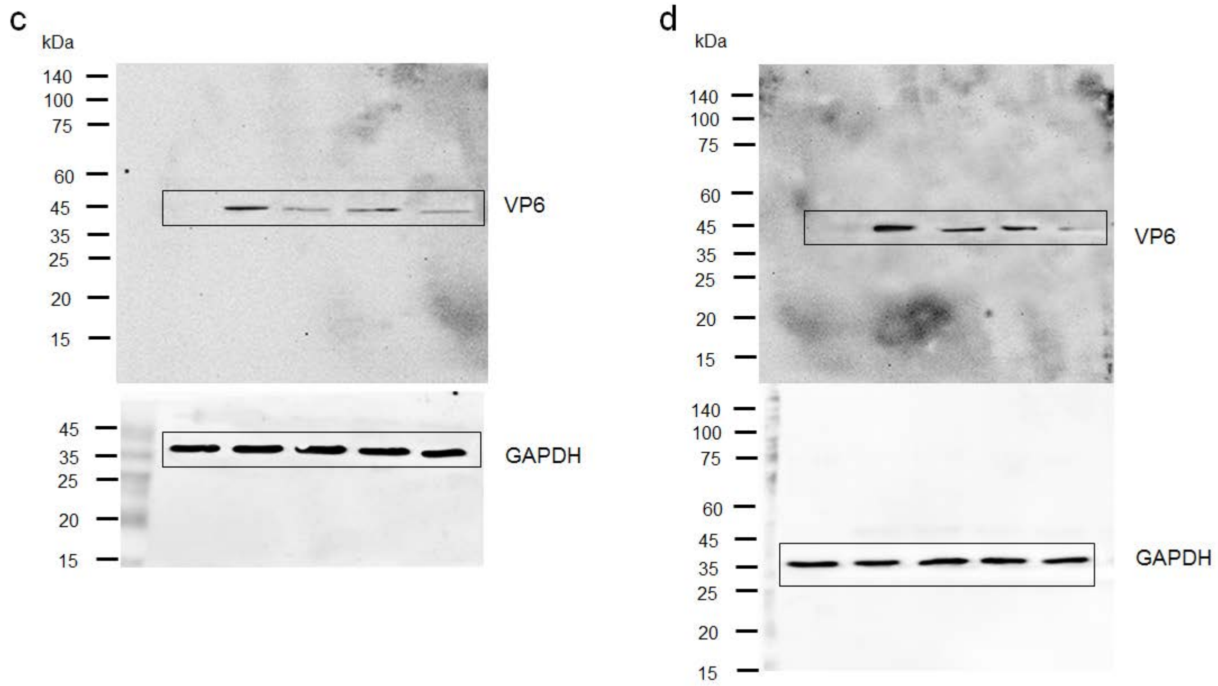

Figure S2 a and b.

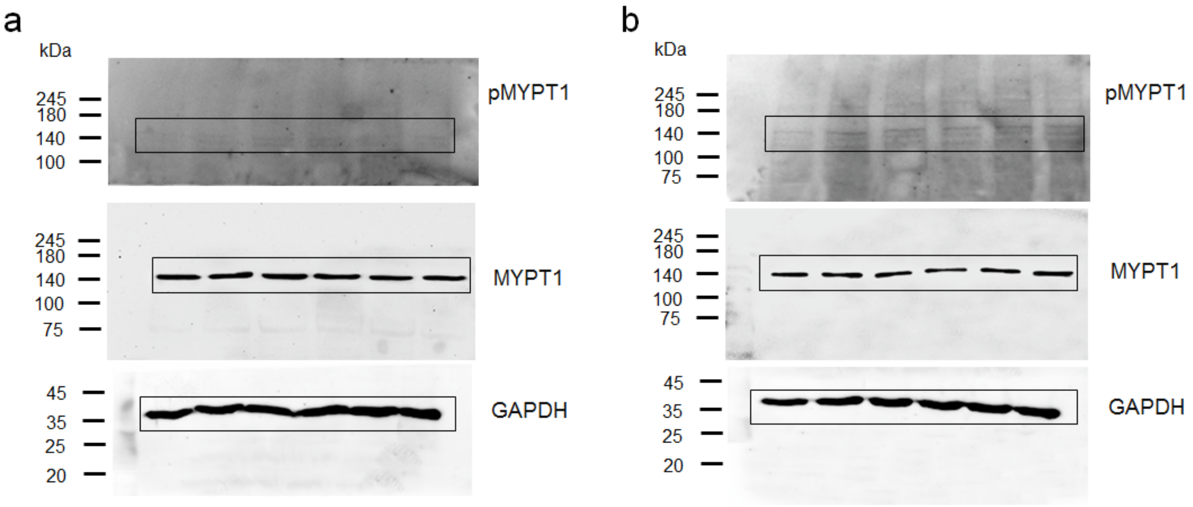

Figure S2 c and d.

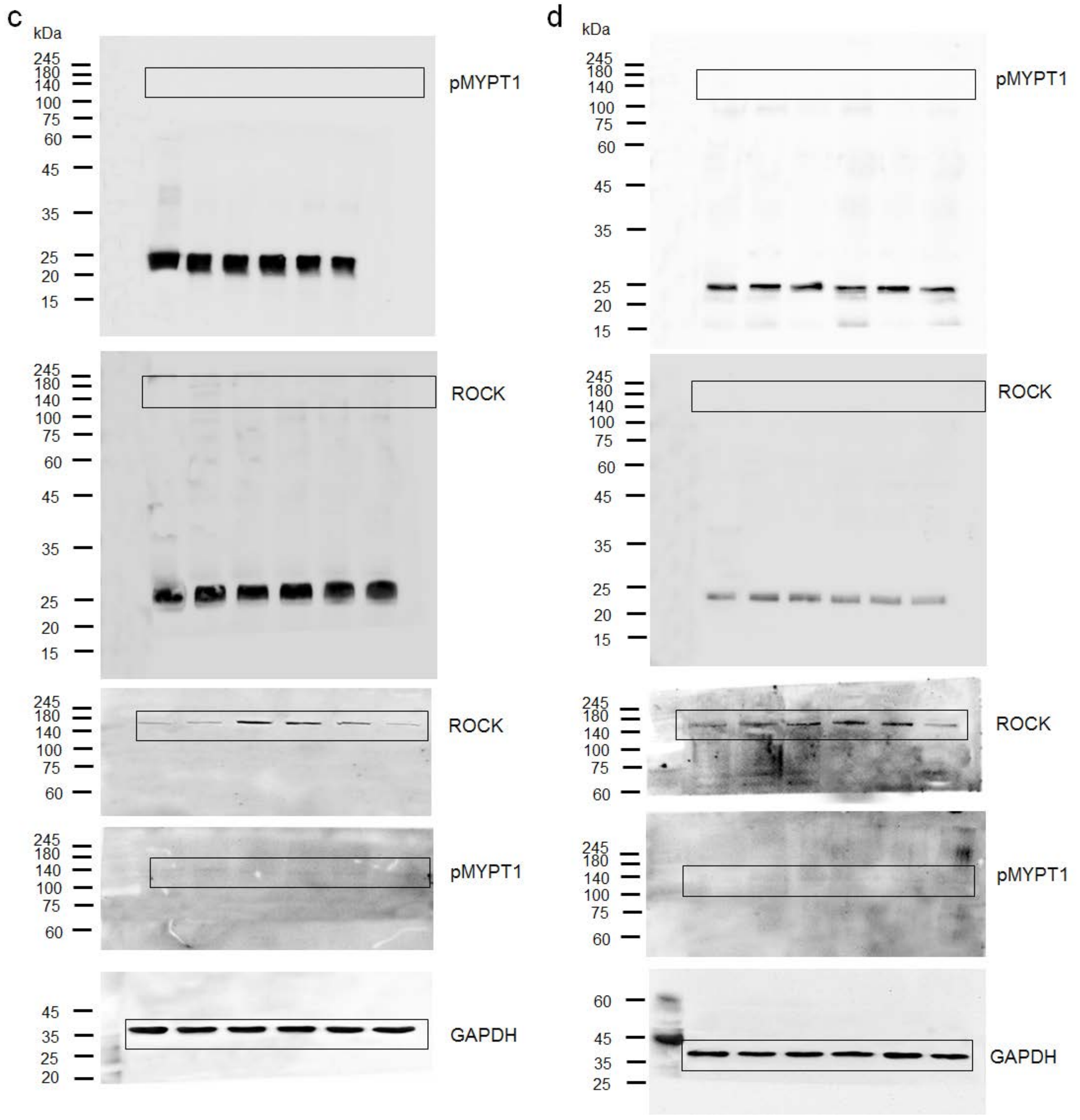

Figure S3.

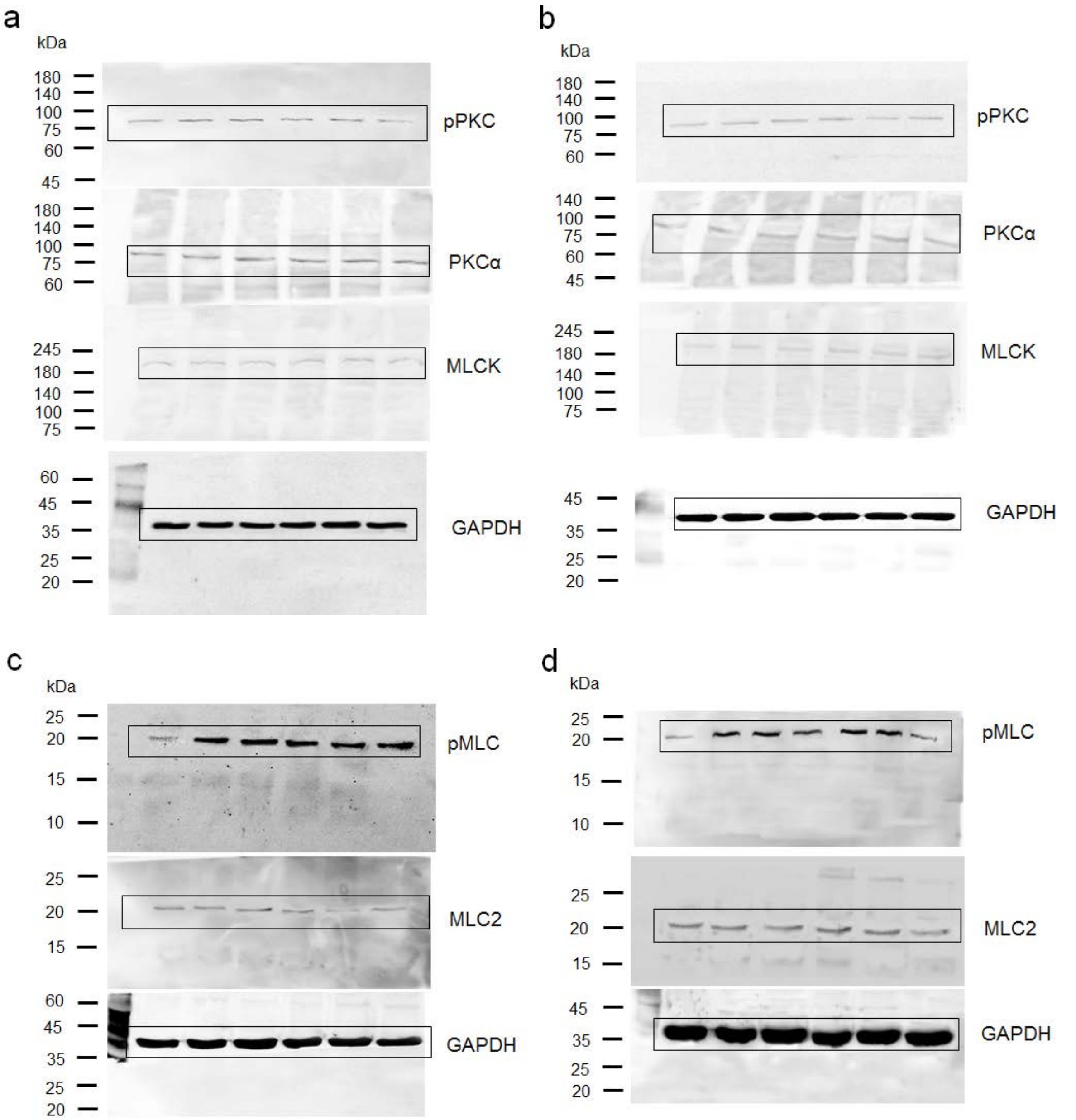
a
kDa
180
140
100
75
60
45
pPKC
180
140
100
75
60
PKCα
245
180
140
100
75
MLCK
60
45
35
25
20
GAPDH
b
kDa
180
140
100
75
60
pPKC
140
100
75
60
45
PKCα
245
180
140
100
75
MLCK
45
35
25
20
GAPDH
c
kDa
25
20
15
10
pMLC
25
20
15
MLC2
60
45
35
25
20
GAPDH
d
kDa
25
20
15
10
pMLC
25
20
15
MLC2
45
35
25
20
GAPDH
